# Identification of Substitutions and Small Insertion-Deletions Induced by Carbon-Ion Beam Irradiation in *Arabidopsis thaliana*

**DOI:** 10.3389/fpls.2017.01851

**Published:** 2017-10-27

**Authors:** Yan Du, Shanwei Luo, Xin Li, Jiangyan Yang, Tao Cui, Wenjian Li, Lixia Yu, Hui Feng, Yuze Chen, Jinhu Mu, Xia Chen, Qingyao Shu, Tao Guo, Wenlong Luo, Libin Zhou

**Affiliations:** ^1^Biophysics Group, Institute of Modern Physics, Chinese Academy of Sciences, Lanzhou, China; ^2^College of Life Sciences, University of Chinese Academy of Sciences, Beijing, China; ^3^College of Life Sciences and Technology, Gansu Agricultural University, Lanzhou, China; ^4^National Key Laboratory of Rice Biology, Institute of Crop Sciences, Zhejiang University, Hangzhou, China; ^5^National Engineering Research Center of Plant Space Breeding, South China Agricultural University, Guangzhou, China

**Keywords:** *Arabidopsis thaliana*, carbon-ion beam (CIB) irradiation, mutation, molecular spectrum, small INDELs, substitutions, whole genome-wide re-sequencing

## Abstract

Heavy-ion beam irradiation is one of the principal methods used to create mutants in plants. Research on mutagenic effects and molecular mechanisms of radiation is an important subject that is multi-disciplinary. Here, we re-sequenced 11 mutagenesis progeny (M3) *Arabidopsis thaliana* lines derived from carbon-ion beam (CIB) irradiation, and subsequently focused on substitutions and small insertion-deletion (INDELs). We found that CIB induced more substitutions (320) than INDELs (124). Meanwhile, the single base INDELs were more prevalent than those in large size (≥2 bp). In details, the detected substitutions showed an obvious bias of C > T transitions, by activating the formation of covalent linkages between neighboring pyrimidine residues in the DNA sequence. An A and T bias was observed among the single base INDELs, in which most of these were induced by replication slippage at either the homopolymer or polynucleotide repeat regions. The mutation rate of 200-Gy CIB irradiation was estimated as 3.37 × 10^−7^ per site. Different from previous researches which mainly focused on the phenotype, chromosome aberration, genetic polymorphism, or sequencing analysis of specific genes only, our study revealed genome-wide molecular profile and rate of mutations induced by CIB irradiation. We hope our data could provide valuable clues for explaining the potential mechanism of plant mutation breeding by CIB irradiation.

## Introduction

The heavy-ion beam is an effective and unique mutagen that can induce mutations at a high rate and on a broad spectrum. It has been widely used in the mutation breeding of plants and microbes because of their distinct physical and biological advantages. The most important physical aspect of the heavy-ion beam is the greater linear energy transfer (LET) than X-rays, gamma rays, and electrons. LET represents the energy deposition of ionizing radiations on their per unit track. This high concentration of deposited energy can cause more severe damage to the target biomolecules, such as, proteins, membrane lipids, and nucleic acids. Hence, the heavy-ion beam provides a higher relative biological effectiveness (RBE) than the low LET radiation (Tanaka et al., 2010; Kazama et al., 2011; Nagata et al., 2016; Zhou et al., 2016). For plant mutation breeding, various mutant populations have been generated by heavy-ion beam irradiation: for instance, those of *Arabidopsis thaliana* (Tanaka et al., 1997, 2002), *Lotus japonicus* (Oka-Kira et al., 2005; Luo et al., 2016), Rice (*Oryza sativa* L.) (Ishikawa et al., 2012; Phanchaisri et al., 2012; Morita et al., 2017), Petunia (*Petunia hybrid*), Hase et al., 2010), *Tricyrtis hirta* (Nakano et al., 2010), Chrysanthemum (Matsumura et al., 2010), Wandering Jew (He et al., 2011), Geranium (Yu et al., 2016), and so on.

The creation of such mutations is an essential resource tool to better link phenotype screening, gene isolation, and function mining, in both forward and reverse genetics. DNA, carrying the genetic information, was originally thought as the main target of radiation. The radiation-induced DNA damage involved direct injury caused by thermal, dynamic, and ionization, as well as the indirect injury caused by the cytotoxic reaction to energy deposition (Ravanat et al., 2001; Kovacs and Keresztes, 2002; Alizadeh et al., 2013; Tokuyama et al., 2015). The mutagenic effects and molecular characteristics of mutations induced by heavy-ion beam are important in two respects: (1) For choosing the most suitable kinds of ionizing radiation; and (2) For elucidating the molecular profile and rate of mutations induced by heavy-ion beam at DNA level. Together, they form an important subject that is multi-disciplinary, such as radiobiology, molecular biology, genetics, biochemistry, etc. In the last century, the characterization of mutations induced by radiation was done by focusing on the phenotype, chromosome aberration, genetic polymorphism or sequencing analysis of specific genes only (Shikazono et al., 2005; Kazama et al., 2011; Hase et al., 2012; Hirano et al., 2012; Yan et al., 2014). However, whether restricted by detecting technology or by high operating costs, we still lack the investigation at whole genome level.

In response to the urgent demands of genome-wide studies, next generation sequencing (NGS) [also called the high-throughput sequencing (HTS)] techniques (Schuster, 2008; Shendure and Ji, 2008)—such as, Illummia/Solexa, Roche/454, and AB/SOLiD—were rapidly developed to enable the fast sequencing and mutation scanning for genomic mutation identification, species evolution, genetic diseases, etc. (Uchida et al., 2011; Belfield et al., 2012; Bolon et al., 2014; Xia et al., 2015; Zhou et al., 2015; Tiwari et al., 2016; Lehrbach et al., 2017; Tao et al., 2017). For instance, using NGS techniques, mutations induced by ethylmethane sulphonate (EMS) were found to be mainly consist of G/C–A/T transitions (Uchida et al., 2011). An EMS-induced causal mutation in *CTR1* required for boron-mediated root development by low-coverage genome re-sequencing in *A. thaliana* was successfully identified though association of re-sequencing and rough map-based cloning (Tabata et al., 2013). An efficient protocol, based on the NGS, to map and identify EMS-induced mutations in *Caenorhabditis elegans* were reported in 2017 (Lehrbach et al., 2017). Moreover, results obtained from NGS techniques have shown that the variations induced by radiation were, to some extent, different from the publications which have been previously reported. Taking the fast neutron exposure for instance: it was deemed to predominantly induce large deletions in size more than a kilo base in *A. thaliana* by the southern blot analysis of the restricted loci. However, a re-sequencing analysis of six mutants derived from fast neutron exposure indicated that the proportion of substitutions prevails over insertion-deletion (INDELs) mutations and that small deletions were more common than large fragment deletions (Bruggemann et al., 1996; Belfield et al., 2012; O'Rourke et al., 2013). Therefore, utilizing the NGS techniques would complement and complete our knowledge of the actual nature of mutations induced by ionizing radiation. The corresponding algorithm tools, such as, the Sequence Alignment/Map (SAM) (Li et al., 2009), Bowtie (Langmead et al., 2009), the SHORE pipeline (Schneeberger et al., 2009), and VarScan (Koboldt et al., 2009, 2012), have been rapidly developed for processing big datasets obtained by NGS. Specifically, the flow of the Illummia/Solexa, one of the most mainstream NGS platforms, is as the following: (1) fragment the genomic DNA; (2) perform end-joining, followed by adding A to 3′ end; (3) ligation of the Solexa adapter; (4) recover and purify the target fragment, then amplify it via PCR; (5) cluster amplification and sequencing-by-synthesis reads obtained by sequencing, (6) data processing. For whole genome re-sequencing of model organisms, the clean reads are mapped against the reference genome (associated with bioinformatics analysis). The genomic mutations (i.e., the sequence and structure variants) are then called by using the corresponding algorithm tools.

In the current study, we re-sequenced 11 mutagenesis progeny (M3) *A. thaliana* lines—nine mutants with visible and heritable traits, and two M3 mutagenesis progeny with inconspicuous phenotypes—derived from carbon-ion beam (CIB) irradiation by using the Illumina sequencing platform. Focusing on substitutions and small INDELs, we revealed the genome-wide molecular profile and rate of mutations induced by CIB irradiation in *A. thaliana*.

## Materials and methods

### Irradiation of CIB and mutant screening

The seeds of laboratory wild type *A. thaliana* (Lab-WT)—Columbia genetic background—were exposed to ^12^C^6+^ ions at 200 Gy (energy, 43 MeV/nucleon; average LET within samples, 50 keV/μm) generated by the Heavy Ion Research Facility in Lanzhou (HIRFL) at the Institute of Modern Physics, Chinese Academy of Sciences (IMP-CAS). Methods of plant growth and mutation screening were described in a previous study (Yan et al., 2014). Nine mutant lines (M3) that displayed visible and heritable traits (C7, C116, C197, C352, C357, C541, C600, C828, C941), as well as two mutagenesis progeny (M3) lines which with inconspicuous mutant phenotypes (C1001, C1322), along with the Lab-WT line were chosen for whole genome re-sequencing.

### Whole genome re-sequencing

Young leaves were collected from the Lab-WT line and 11 M3 lines derived from CIB. Genomic DNA was extracted by using the routine protocol of CTAB (cetyltrimethylammonium bromide). The genomic re-sequencing was performed by the IIIumina HiSeq^TM^ 2500 system (Illumina, Inc.; San Diego, CA, USA) at the Biomarker Technologies Company (Beijing, China). Raw data were filtered following the standard process of Illumina, and the clean data obtained were then mapped onto the reference genome of Col-0 (TAIR10, http://www.ncbi.nlm.nih.gov/assembly/GCF_000001735.3) by using the Burrows-Wheeler Alignment tool (http://bio-bwa.sourceforge.net/bwa.shtml, v.0.7.15; Li and Durbin, 2009) and SAMtools (http://www.htslib.org/workflow/#mapping_to_variant, v.1.3.1; Li et al., 2009). The average depth of the re-sequenced lines had a 23–33-fold range, while those reads with a depth >10-fold accounted for up to 98.87% of the total reference genome, on average (Table S1).

### Identification of the variants

The VarScan 2 algorithms (v.3.9, http://varscan.sourceforge.net) were used to read the SAMtools mpileup output, and the substitutions and small INDELs were called with settings described below:

Min coverage: Minimum read depth at a position to make a call is eight;

Min reads2: Minimum supporting reads at a position to call variants is two;

Mmin-var-freq: Minimum variant allele frequency threshold is 0.01;

*P*-value: Default *p*-value threshold for calling variants is 0.99.

The true candidate mutations induced by CIB were screened as followed steps:

1. Following the VarScan 2 algorithms, to filter the background mutations in the Lab-WT line—and to identify the particular mutations of each mutagenesis progeny—only those mutation sites with a variant allele frequency by read count were between 25–100% in a sample, while 0–10% in other samples were reserved; those variants with a variant allele frequency of ≥75% were called homozygous. Against this, the sites shared by the 12 sequenced lines were called as the background mutations.
2. Although the filtering standard of step (1) was effective at reducing the rate of false positives, to ensure the accuracy of mutation detection, the candidate mutations derived from step (1) of each sequenced line were visually confirmed in the Integrative Genomics Viewer (http://software.broadinstitute.org/software/igv/, v.2.3).

### Mutation annotation

Variant annotation and predicted effects were performed by the SnpEff toolbox (http://snpeff.sourceforge.net/index.html, v.4.2).

### Verification of the mutation sites by sanger sequencing

The specific primers of mutation sites obtained by re-sequencing were designed by PRIMER3 (http://bioinfo.ut.ee/primer3-0.4.0/). The primers used for PCR are P1 (5′-TTTGCTGTTGGTAGTGTGCC-3′, 5′-TGGAAGTAGAGTGCAGCGAT-3′), P2 (5′-TATGCTTGCACGTTTGCTTC-3′, 5′-GGCTTGAGAGTTGGGTGAAC-3′), P3 (5′-CACTTCTTTTCCCCAACGGT-3′, 5′-TACATGCTTGCATCACCCAC-3′), P4 (5′-GACGACCTCCCAGATATGCC-3′, 5′-GGAGATTGTGGGGTCCCAAG-3′), P5 (5′-CAGGAGCAACTTACCAACGG-3′, 5′-GAACAATACAGGTGGCGTGG-3′), P6 (5′-TGTGATCAAGAGTGTCGGCT-3′, 5′-ACGTCAAAACTTCCTCCCCA-3′) and P7(5′-CCCCTGATAAGTTGCGTTTAAGT-3′, 5′-ACTTGATGACATGGGAGGCA-3′). Genomic DNA was extracted by using the routine protocol of CTAB. PCR amplification was performed with an initial denaturation step at 95°C for 5 min, followed by 38 cycles at 94°C for 30 s, 58°C for 30 s, and 72°C for 30 s, with a final extension step at 72°C for 10 min. The PCR products were then detected by electrophoresis on a 1.5% agarose gel; only the qualified PCR products were used for Sanger sequencing.

### Data availability

The whole-genome sequencing data reported in this study have been deposited in the Genome Sequence Archive (Genomics, Proteomics & Bioinformatics, 2017) in BIG Data Center (Nucleic Acids Res, 2017), Beijing Institute of Genomics (BIG), Chinese Academy of Sciences, under accession number CRA000464 that is publicly accessible at http://bigd.big.ac.cn/gsa.

## Results

### CIB irradiation and mutagenesis progeny

**Figure 1:**
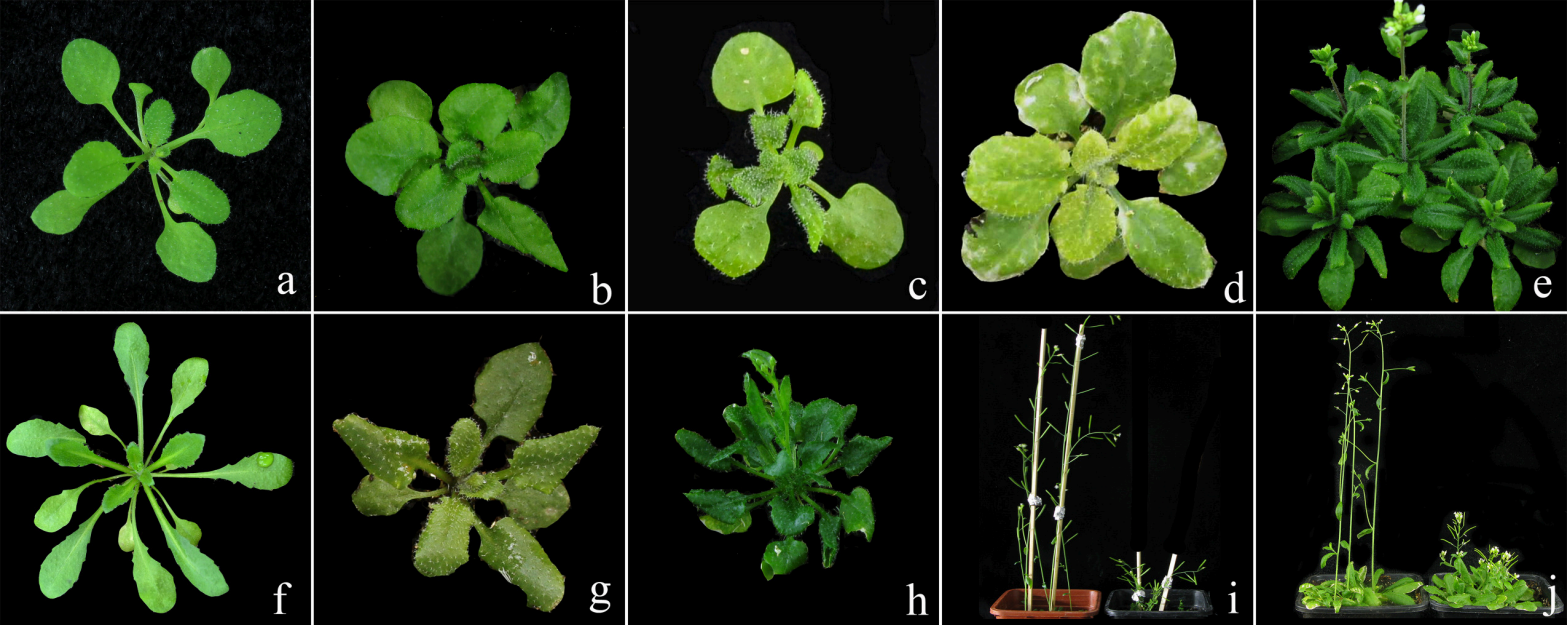
Phenotypes of the nine re-sequenced lines that had stable mutation traits induced by CIB irradiation. **(a)**: wild type (ecotype Col); **(b–j)**: mutagenesis progeny lines in the corresponding order of C7, C197, C352, C357, C600, C828, C941, C116 and C541.

The Lab-WT *A. thaliana* seeds were exposed to CIB irradiation with the aim (though not the focus of the present study) of constructing a comprehensive carbon-ion-induced mutation resource collection for *A. thaliana*. The M2 seedlings were screened for abnormal phenotypes by comparison with the Lab-WT; meanwhile, several lines lacking obvious mutant traits were also reserved. In total, more than 1,000 independent plants were isolated. Our subsequent analyses were based on nine mutant lines (M3) that displayed visible and heritable traits (i.e., C7, C116, C197, C352, C357, C541, C600, C828, C941), as well as two mutagenesis progeny (M3) lines which with inconspicuous mutant phenotypes (i.e., C1001, C1322), together with the Lab-WT line were chosen for whole genome re-sequencing. The phenotypes of the nine stable lines are shown in Figure 1.

### Mutations shared by the 12 sequenced lines

**Figure 2:**
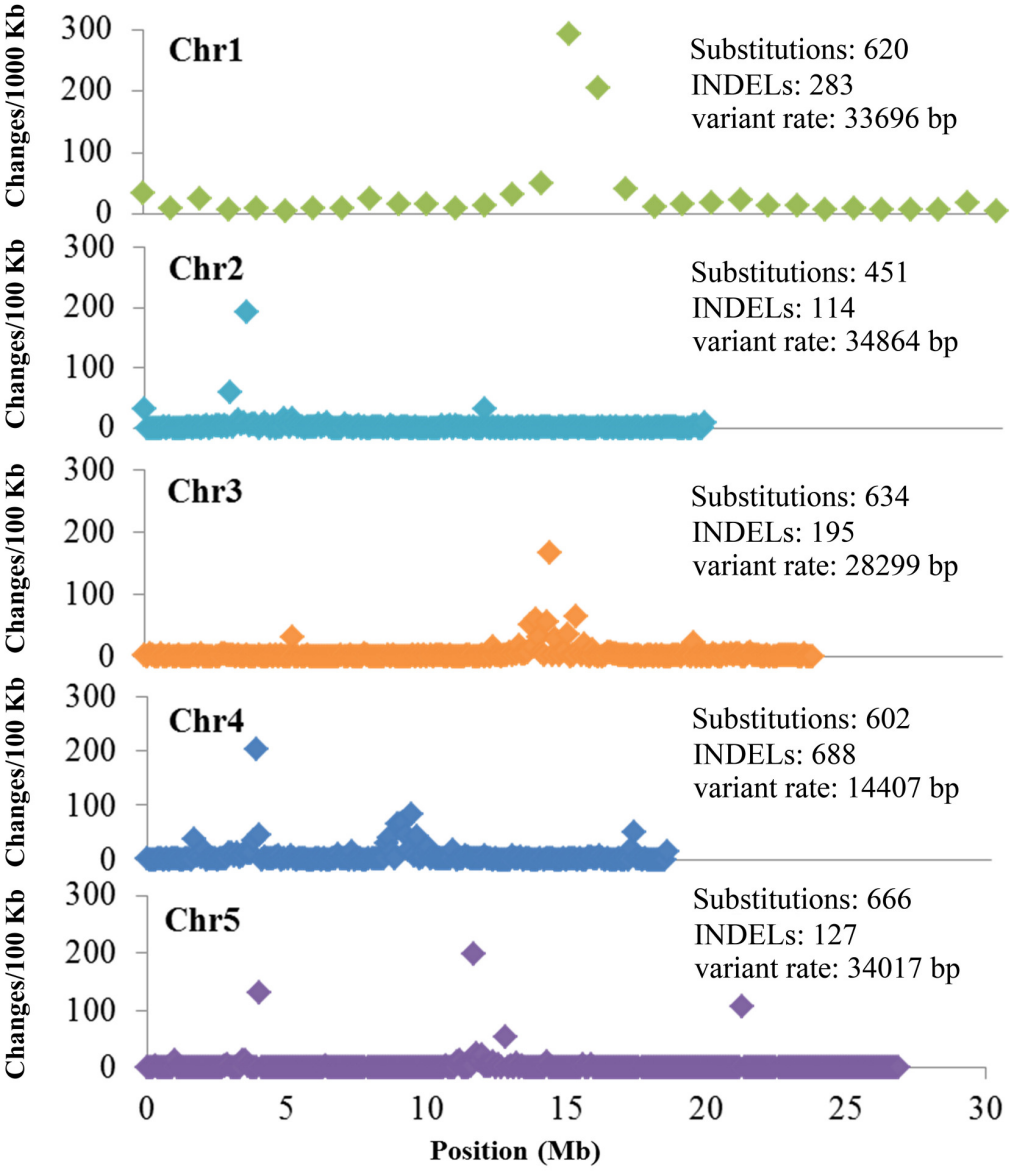
Background mutations shared by the 12 re-sequenced *Arabidopsis thaliana* lines. Shown are the density and variant rate of the single base substitutions and small INDELs that were shared by 12 re-sequenced lines across each chromosome.

DNA sequence data from a single Lab-WT line and other 11 mutagenesis progeny lines were aligned to the *A. thaliana* Information Resource (TAIR 10) (http://www.arabidopsis.org) Col-0 reference genome by using BWA, Samtools, and VarScan 2. The mapping results for the mutant sequencing reads for each line are shown in Table S1. Before the variants analysis, we surmised that there were a number of substitutions and INDELs which might be the background discrepancies that originally existed between the Lab-WT and Col-0 reference genomes. To identify those variations truly induced by CIB irradiation, it was reasonable to rule out these background discrepancies firstly. Analyzed by group, since the 11 mutagenesis progeny lines were in the same background (Columbia), the variants shared by the 12 sequenced lines were considered as the background mutations. On this premise, a total of 2,973 substitutions and 1,407 INDELs were identified (Figure 2).

**Figure 3:**
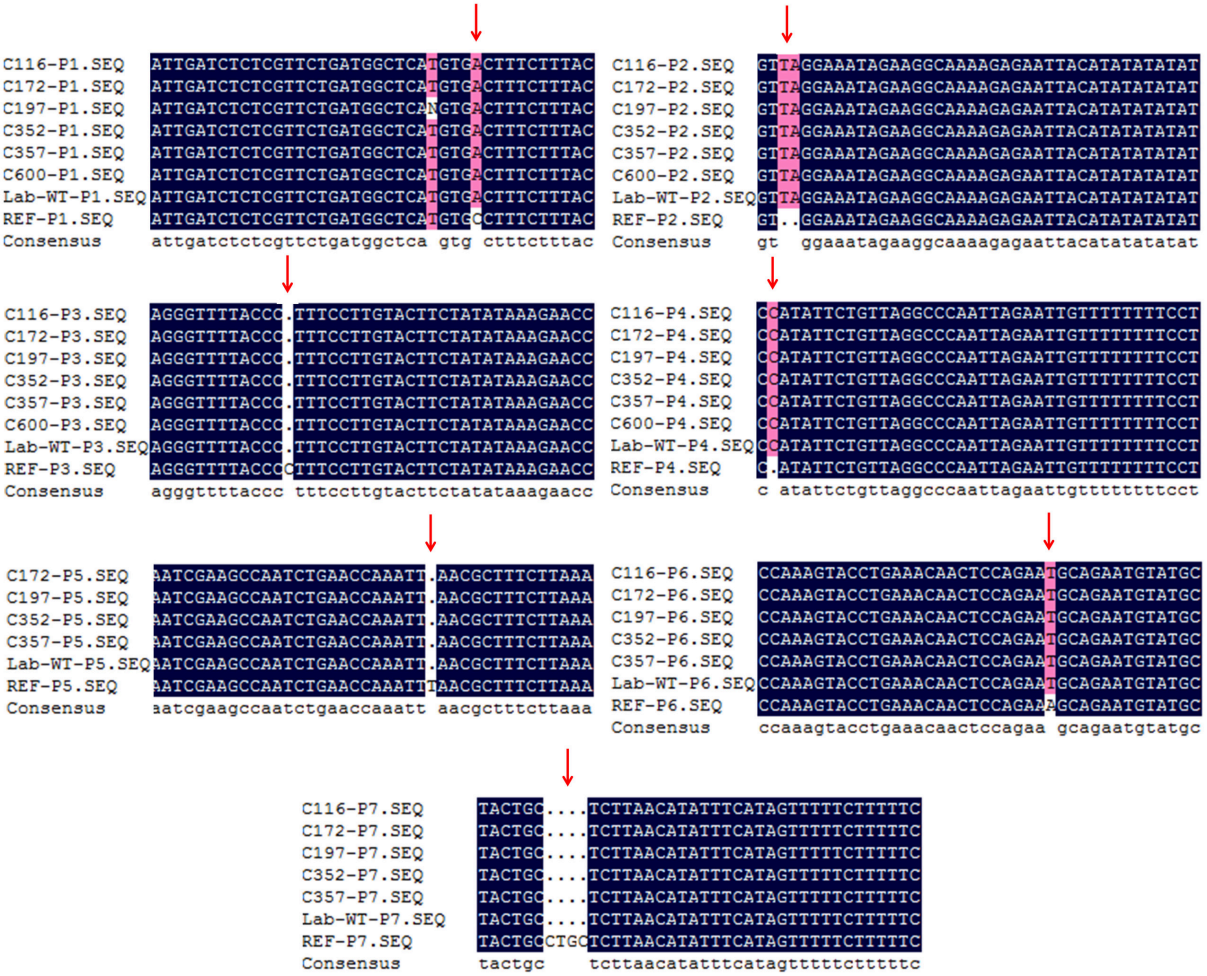
Verification of background mutations by Sanger sequencing. The REF denotes the Col-0 reference genome. The sequence alignment of different primers (P1–P7) from C116, C172, C197, C352, C357, Lab-WT and reference were shown with different colors according to their sequence homology. Pink highlights the sequence which are different from the reference genome, the dot indicates deletions in the corresponding position.

To verify whether the background mutations were indeed correct, seven sites shared by the 12 sequenced lines were selected for Sanger sequencing. To increase the credibility, another M3 line (C172), one not re-sequenced by NGS, was also included. The Sanger sequencing results indicated that those sites obtained by NGS were indeed common background mutations in our Lab-WT (Figure 3). These background mutations were unevenly distributed in the Lab-WT genome: the highest mutation number was observed on chromosome 4, with a change rate of one per 14,407 nucleotide base pairs along the genome. Interestingly, substitutions and INDELs tended to occur in the peri-centromeric regions of each chromosome as clusters, especially in chromosome 1. This result is basically consistent with the spontaneous mutations in *A. thaliana* in previous report (Ossowski et al., 2010), although the mechanism of such a mutational bias remains unexplored.

### Distribution and rates of mutations induced by CIB irradiation

**Figure 4:**
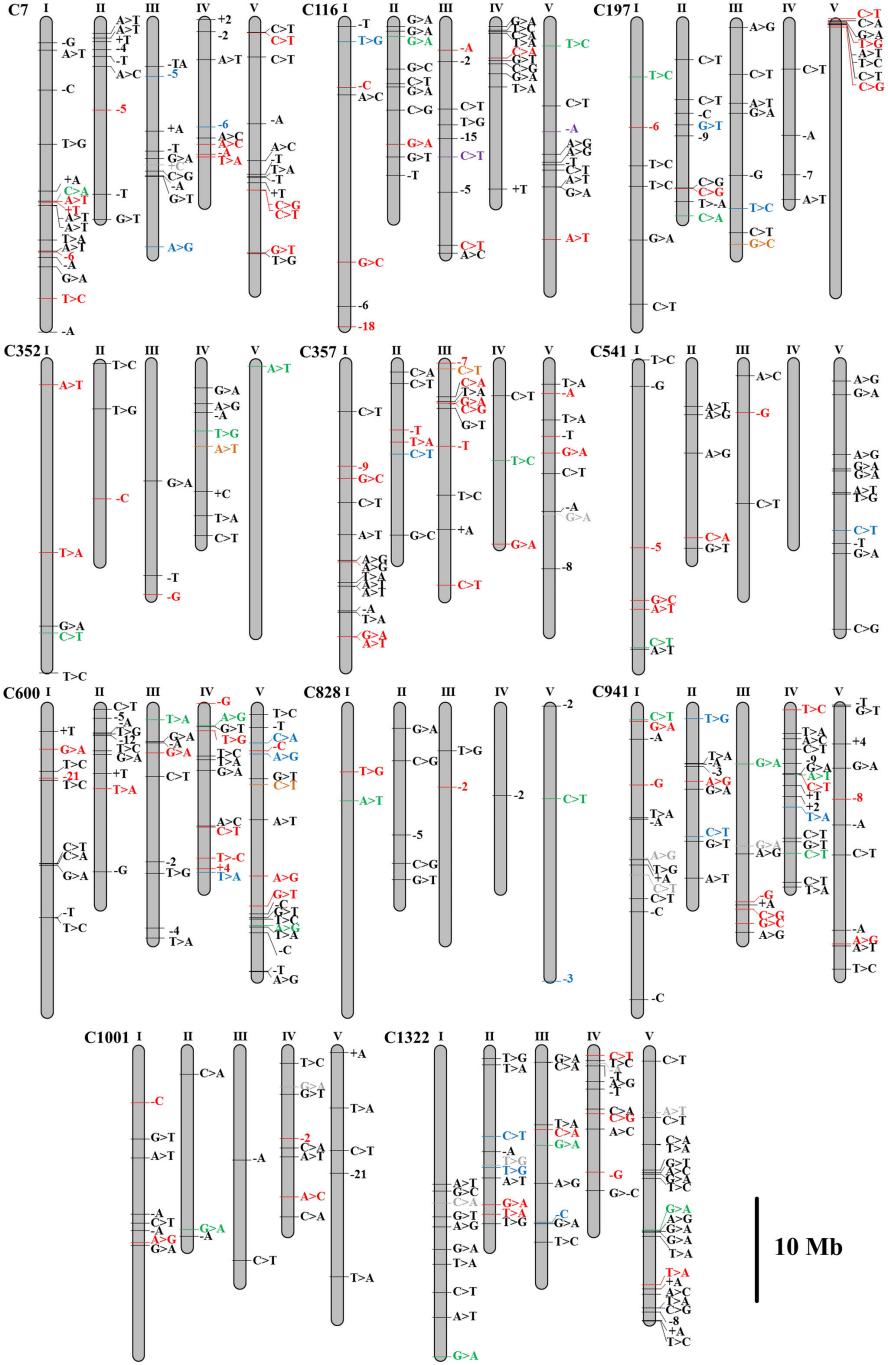
Distributions of the substitutions and small INDELs across chromosomes in the genomes of 11 mutagenesis progeny lines (M3) of *Arabidopsis thaliana*. Single-base INDELs are indicated by base-designating letters, with a preceding minus sign (deletion) or plus sign (insertion). Multiple-base INDELs are indicated by a minus or plus sign with the number of deleted or inserted bases. Individual colors distinguish the possible mutation effects: missense, frame shift, in-frame deletion, stop gained/lost (red); synonymous (green); UTR (blue); splice site region, intron (orange); intergenic region (gray); up downstream regions (black); non-coding exon (purple).

**Figure 5:**
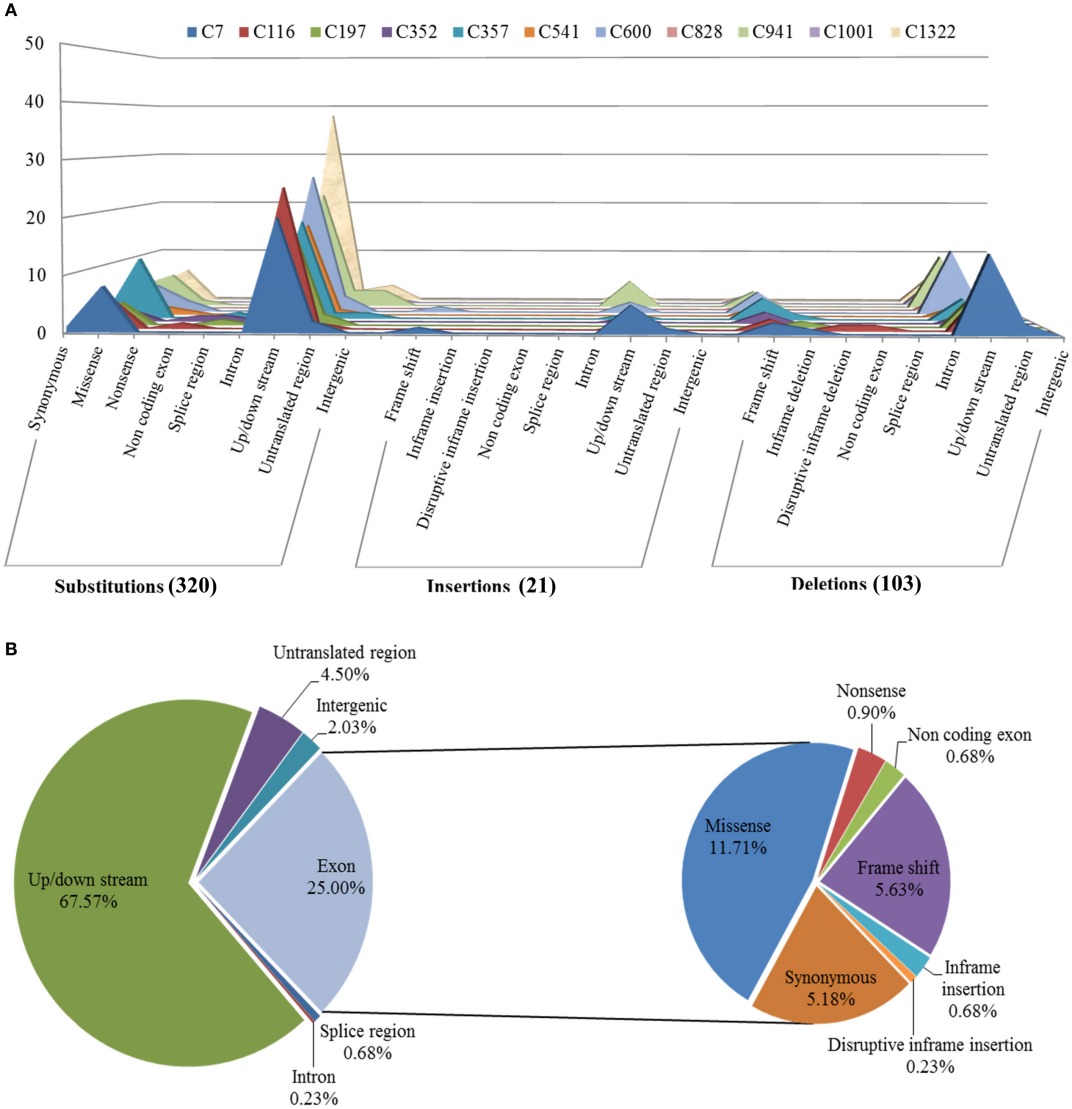
Annotation of mutations induced by CIB irradiation in genomes of 11 mutagenesis progeny lines (M3) of *Arabidopsis thaliana*. **(A)** Mutations comprehensively inferred by BWA, SAMtools, VarScan 2, as well as their distributions among functional classes and mutation effects in each CIB-irradiated M3 plant line. **(B)** Overall distribution of mutations induced by CIB irradiation at the whole genomic level in *Arabidopsis thaliana*.

To identify the exclusive mutations of each mutagenesis progeny line, combining the mutation calling by VarScan 2 and the visual confirmation by the Integrative Genomics Viewer (IGV), a total of 320 substitutions and 124 INDELs were detected, and the ratio of substitutions to INDELs was calculated as 2.58:1 (Figures 4, 5A). The number of variations per line ranged from 20 to 62, and their distribution across the chromosomes in each of the 11 lineages is shown in Figure 4. However, no remarkable regularity in variant rate of single base substitutions and small INDELs across each chromosome were found in the 11 re-sequenced lines (Table 1). At the genome-wide scale, 67.57% of the 444 detected CIB irradiation-induced mutations occurred in the upstream and downstream regions, 4.50% in the 3′/5′-untranslated regions (UTR), 25.00% in exon, 0.68% in the splice region, 0.23% in intron, and 2.03% in the intergenic region (Figure 5B).

**Table 1:** Variant rate of the single base substitutions and small INDELs across each chromosome in the 11 re-sequenced lines.

**Line**	**Chromosome 1**		**Chromosome 2**		**Chromosome 3**		**Chromosome 4**		**Chromosome 5**	
	**Variants^a^**	**Rate^b^**	**Variants**	**Rate**	**Variants**	**Rate**	**Variants**	**Rate**	**Variants**	**Rate**
C7	17	1789863	9	2188699	10	2345983	8	2323132	13	2075039
C116	7	4346810	10	1969829	9	2606648	10	1858506	10	2697550
C197	6	5071279	9	2188699	8	2932479	4	4646264	8	3371938
C352	5	6085534	3	6566096	3	7819943	8	2323132	1	26975502
C357	14	2173405	6	3283048	11	2132712	3	6195019	9	2997278
C541	7	4346810	5	3939658	3	7819943	0	/	11	2452318
C600	10	3042767	10	1969829	9	2606648	12	1548755	18	1498639
C828	2	15213836	5	3939658	2	11729915	1	18585056	3	8991834
C941	13	2340590	9	2188699	8	2932479	16	1161566	11	2452318
C1001	8	3803459	3	6566096	2	11729915	8	2323132	5	5395100
C1322	10	3042767	10	1969829	9	2606648	11	1689551	22	1226159

a *Variants indicate the number of mutations in each chromosome*.

b *Rate equals to length of chromosome/variants. Length (bp) of chromosome 1 to 5 is 30427671, 19698289, 23459830, 18585056, and 26975502, respectively*.

**Figure 6:**
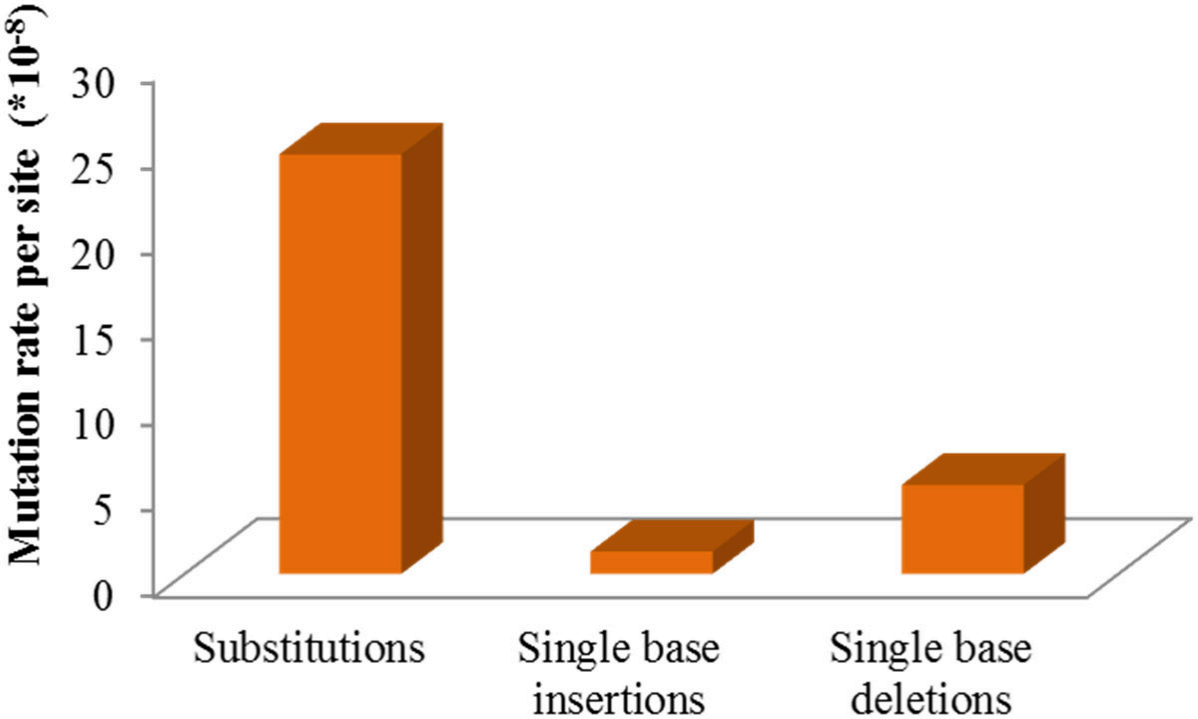
Single nucleotide mutation rates of CIB irradiation on *Arabidopsis thaliana*.

**Figure 7:**
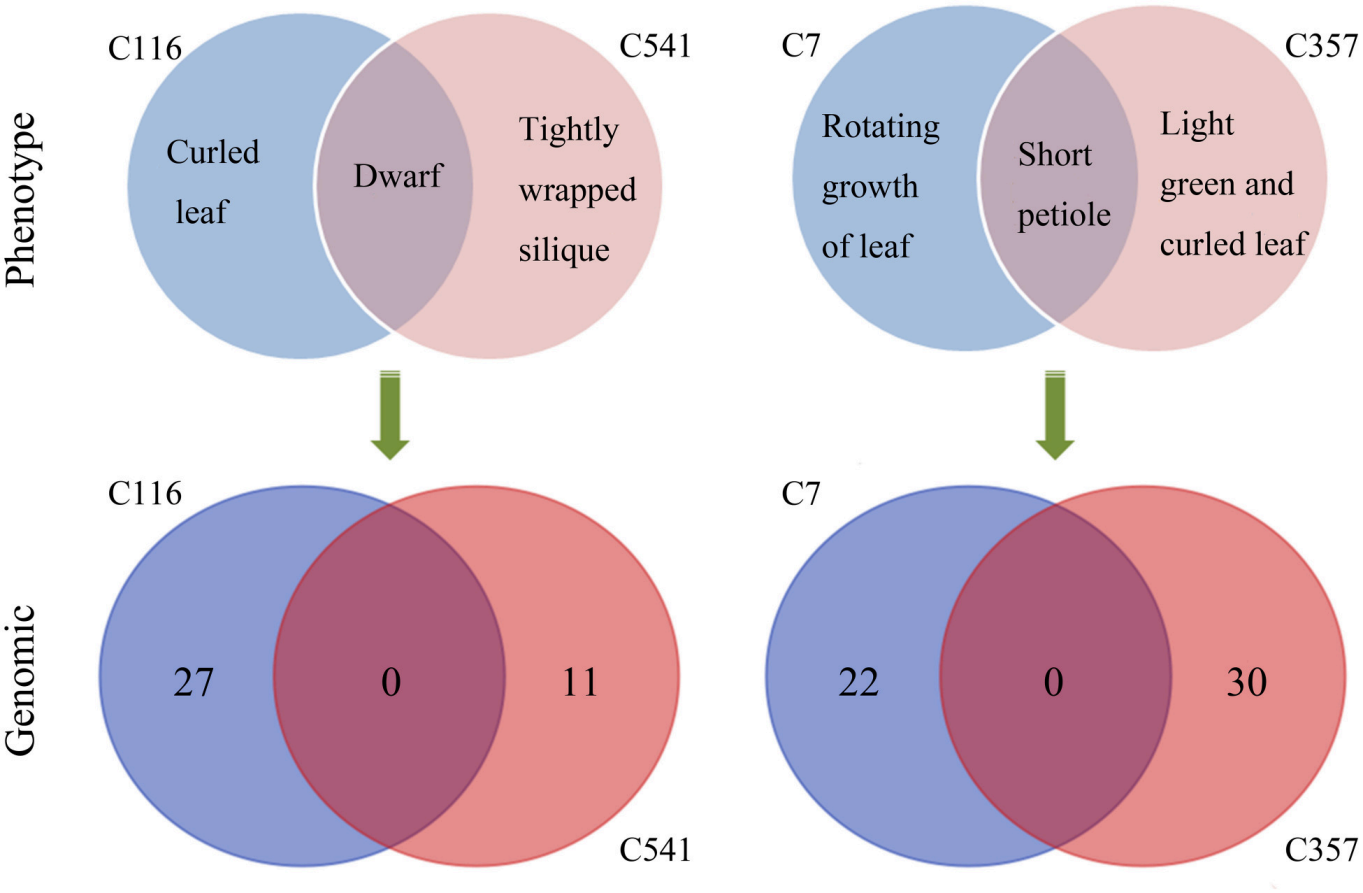
Similarity between the mutant lines with similar phenotypes at the genomic level. The homozygous mutations in M3 were used to detect whether there were any common mutations at the genomic level between the re-sequenced lines sharing similar phenotypes.

Mutations involved in missense, stop gained/lost, frame shift, in-frame deletion, and 3′/5′-UTR, are usually predicted to affect gene function more probably. Classifying all the above sites including both heterozygous and homozygous, 103 mutations located in 97 genes were sorted, and the number of mutated genes affected by irradiation in the M3 lines ranged from 3 to 15 (Table 2 and Table S2). The homozygous mutations in M3 were used to estimate the M1 heterozygous mutation events caused by CIB irradiation followed Mendelian principles (Belfield et al., 2012). The total number of single base substitutions, single base insertions, and single base deletions in M1 of the 11 sequenced lines were 362.67, 18.67, and 69.33, respectively (Table S3). After correcting the spontaneous mutations by using the Col-0 MA line mutation rates (Ossowski et al., 2010), on average the single base mutation rate was estimated to be 3.37 × 10^−7^ per site per genome (Figure 6). The mutation rate of the 200-Gy CIB irradiation was nearly 47-fold that of the spontaneous rate (7.1 × 10^−9^ per site). Since the phenotypes of C7, C357, C116, and C541 were heritable, the homozygous mutations in M3 were used to detect whether there was any common mutations at the genomic level. However, no shared genetic factors were found between C116 and C541, or between C7 and C357. To some extent, this indicated that the CIB irradiation was able to induce mutation in multiple genes which could give rise to similar traits (Figure 7).

**Table 2:** Numbers of genes predicted to occur function changes in the 11 re-sequenced lines.

	**Homozygous**				**Heterozygous**				**Total mutated genes^c^**
**Line**	**3′/5′UTR**	**Non-synonymous^a^**	**Frame shift**	**Disruptive inframe_del^b^**	**3′/5′UTR**	**Non-synonymous**	**Frame shift**	**Disruptive inframe_del**	
C7	2	4	1	0	2	3	3	1	13
C116	0	5	2	1	1	0	0	0	9
C197	0	0	0	0	2	4	0	1	7
C352	0	2	2	0	0	0	0	0	4
C357	1	6	3	0	0	3	1	1	15
C541	0	1	1	0	1	2	1	0	6
C600	1	1	0	0	2	6	5	0	15
C828	0	0	0	0	1	1	1	0	3
C941	2	3	1	0	1	4	2	0	13
C1001	0	1	1	0	0	1	1	0	4
C1322	0	0	0	0	2	6	1	0	9

a *Non-synonymous mutations include those mutations with the effect of missense, stop gained, stop lost, and initiator codon variant*.

b *Disruptive inframe_del indicates that the mutation lead one codon changed, and one or more codons are deleted*.

c *When multiple mutations are located in the same gene in one re-sequenced line, and these mutations are viewed as a single gene-mutated*.

### Single base substitutions induced by CIB irradiation

**Figure 8:**
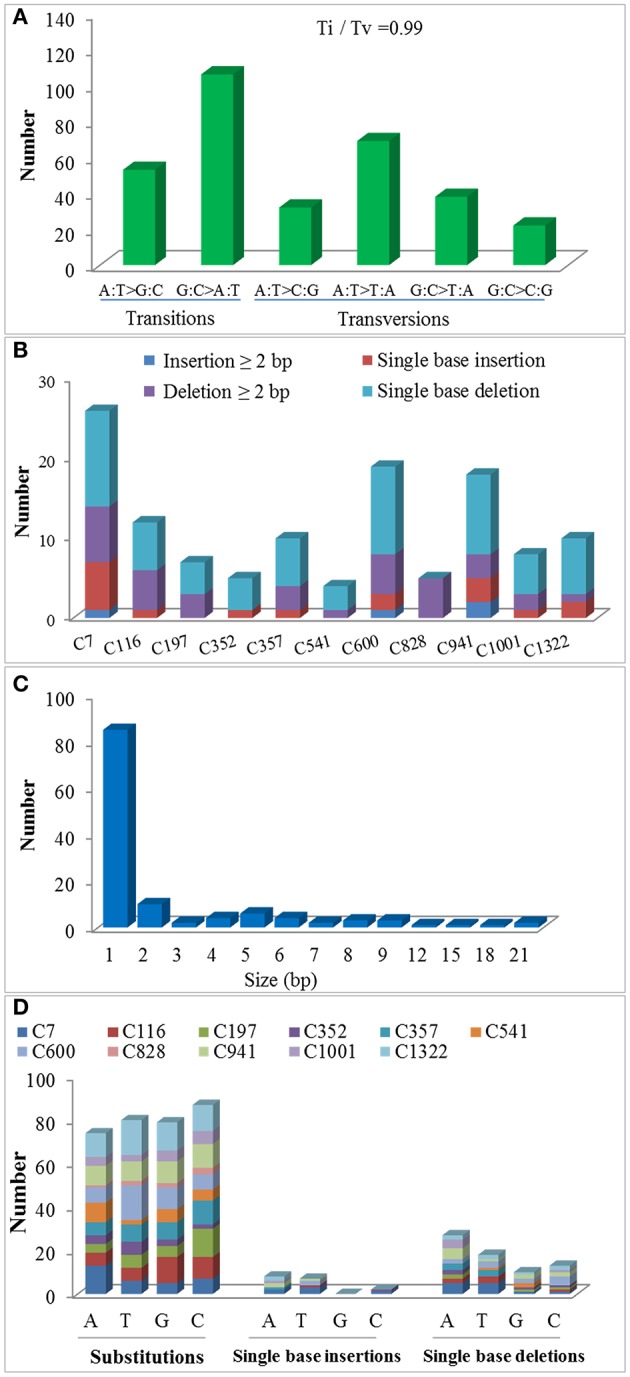
Molecular spectrum of CIB irradiation induced substitutions and small INDELs in the genomes of 11 M3 lines. **(A)** The ratio of transition to transversion (Ti/Tv). **(B,C)** Size distributions of the small INDELs. **(D)** Base bias of CIB irradiation-induced single base mutations.

Exposure to CIB irradiation induced plenty of substitutions. Their type may be classified into two categories: transition (mutations that happen among the same type of bases; e.g., purine > purine or pyrimidine > pyrimidine) and transversion (mutations that happen among the different types of bases; e.g., purine > pyrimidine or pyrimidine > purine). A total of 320 CIB irradiation-induced substitutions were identified and classed, yielding a transition to transversion (Ti/Tv) ratio of 0.99. This indicated that the CIB irradiation was able to induce transitions and transversions at nearly the same frequency (Figure 8A). The G:C > A:T were the most frequently observed substitutions in the re-sequenced mutagenesis progeny lines: 51 of the 320 detected CIB-induced mutations were G > A transitions, 55 were C > T mutations (Table S4). The C > T transitions hold the biggest ratio of substitutions induced by the CIB. By analyzing the flanking DNA sequences of the most prominent substitutions of C > T variations, it was found that 37 of these (67.2%) occurred at the pyrimidine dinucleotide sites (Table 3). In addition, the substitutions seemed more likely to happen at the C base site (87) rather than at other base sites (A, G, and T) (Figure 8D).

**Table 3:** Pyrimidine dinucleotide analyses at C > T substitutions sites in 11 CIB irradiated M3 lines.

**Line**	**Chromosome**	**Loci**	**Mutation**	**Flanking sequence**	**Pyr-Pyr**
C7	5	1496355	C > T	AATT**C**TTGA	T**C** **C**T
C7	5	1454369	C > T	CCTT**C**ACAG	T**C**
C7	5	3829172	C > T	TCAA**C**ATCA	
C7	5	16650819	C > T	GACT**C**TCCA	T**C** **C**T
C116	2	6339827	C > T	GGCT**C**TCAA	T**C** **C**T
C116	3	8857172	C > T	AGTA**C**GCGT	
C116	3	13445052	C > T	CAGT**C**GTTG	T**C**
C116	3	21948981	C > T	TTCT**C**CACT	T**C** **C**C
C116	5	8571804	C > T	CCTT**C**AGGT	T**C**
C116	5	14749784	C > T	ACAA**C**TGTC	**C**T
C197	1	27664800	C > T	TTTG**C**TTTT	**C**T
C197	2	7777170	C > T	CTCT**C**AGCT	T**C**
C197	2	3980109	C > T	CGGG**C**GATG	
C197	3	5423074	C > T	TTAA**C**AGGC	
C197	3	20612671	C > T	AATA**C**ACGT	
C197	4	4989792	C > T	ACGC**C**CAAT	C**C** **C**C
C197	5	12293490	C > T	CTCT**C**ATGT	T**C**
C197	5	5133036	C > T	ATTG**C**AACA	
C352	1	26491174	C > T	TTTT**C**AGTA	T**C**
C352	4	17139849	C > T	TCTA**C**ATAT	
C357	1	5147165	C > T	ATAT**C**AGAT	T**C**
C357	1	13896252	C > T	TGTT**C**TCAC	T**C** **C**T
C357	2	2382455	C > T	ACAC**C**AGGA	C**C**
C357	2	9045207	C > T	TGCA**C**AACA	
C357	3	959295	C > T	AGTT**C**TTGC	T**C** **C**T
C357	3	21754406	C > T	GAAG**C**ATTT	
C357	4	3608407	C > T	ACTA**C**TATC	**C**T
C357	5	11079083	C > T	TGTT**C**AACG	T**C**
C541	1	27821135	C > T	TAGG**C**AAGA	
C541	3	13945744	C > T	TGTT**C**CGCG	T**C** **C**C
C541	5	16565496	C > T	GTTT**C**TTCT	T**C** **C**T
C600	1	15506551	C > T	ACTG**C**AGAG	
C600	2	629920	C > T	CACT**C**TGAT	T**C** **C**T
C600	3	7132293	C > T	GAAA**C**GAGA	
C600	4	12005191	C > T	AACA**C**ATTT	
C600	5	7896135	C > T	CTGA**C**ATTC	
C828	5	9262872	C > T	AGCA**C**ATTT	
C941	1	1654865	C > T	AAAT**C**AGCA	T**C**
C941	1	16595744	C > T	AACT**C**TAAA	T**C** **C**T
C941	1	18884415	C > T	GAAA**C**AAGG	
C941	2	12645936	C > T	TCTT**C**GCTC	T**C**
C941	4	4481201	C > T	TTAA**C**TTTT	**C**T
C941	4	6924288	C > T	AGCA**C**CAGC	**C**C
C941	4	13051872	C > T	ATTA**C**TTCA	**C**T
C941	4	14525988	C > T	GATT**C**TACT	T**C** **C**T
C941	4	17299998	C > T	TAAA**C**AGTT	
C941	5	14679245	C > T	CCAA**C**TCCT	**C**T
C1001	1	17132595	C > T	TTAT**C**ATGT	T**C**
C1001	3	20688685	C > T	AAAA**C**CTAA	**C**C
C1001	5	10126078	C > T	ATAC**C**ACCT	C**C**
C1322	1	23839536	C > T	AATG**C**GTTT	
C1322	2	8613063	C > T	CTCT**C**AATA	T**C**
C1322	4	980039	C > T	GAAT**C**CATC	T**C** **C**C
C1322	5	1431634	C > T	TTTC**C**TTTC	C**C** **C**T
C1322	5	6887544	C > T	GGTT**C**TGTT	T**C** **C**T

*The C base occurring in the substitution sites are in bold and underlined*.

### Small INDELs induced by CIB irradiation

For a long time, deletions were considered the main mutation type induced by heavy-ion beam irradiation, or at least had a similar frequency to substitutions, judging from the sequencing analysis of specific genes only. In present study, at a genome-wide scale, 124 small INDELs were detected, of which 103 (83.06%) were deletion mutations, whereas only 21 (16.94%) were insertions (Figure 8B). Among the 103 small deletions, 35 (33.98%) multiple bases deletions (≥2 bp) ranging in size from 2–21 bp were detected (Figures 8B,C). In addition, 68 (66.02%) single base deletions were detected as well, which seemed the most common class of deletion mutation. Meanwhile, 17 (80.95%) of the 21 insertions were single base mutations, of which only four (19.05%) has a size ≥2 bp. Interestingly, for the single-base INDELs, we noticed that there was a bias to the A and T bases: 45 of the 68 single base deletions and 15 of the 17 single base insertions were A and T (Figure 8D). To explore the rule of small INDELs occurring, we investigated the flanking sequence of 63 small INDELs in six randomly selected sequenced lines (Table 4). Eight (88.89%) of the nine single base insertions and 29 (82.86%) of the 35 single base deletions, along with 16 (84.21%) of the 19 INDELs that had a size ≥2 bp occurred within or near the homopolymer or polynucleotide repeats.

**Table 4:** Flanking sequences analysis of the small INDELs in six randomly selected re-sequenced lines.

**Line**	**Chromosome**	**Loci**	**INDEL size (bp)**	**Sequence**
C7	1	16803045	+1	AAAAATAATT**A**AAAAACGAAA
C7	1	17871566	+1	GATGAGTCTC**T**TTGACAATGA
C7	2	2330971	+1	CTAATCTCTG**T**TTTTTTTTTT
C7	3	14218519	+1	AAAAACCGAT**C**AGAAGAATTC
C7	3	10963641	+1	AACATGTGGC**A**AAAATAAATT
C7	5	15938548	+1	TAAAGTTAGA**T**TTTTTATTTT
C116	4	16566783	+1	TTTTTTTTTC**T**TTTTTTTTTT
C352	4	12840961	+1	TTTAAAAAAA**C**TATTCACAAT
C357	3	16396219	+1	CAAACTTTCT**A**AAAACTCAGC
C7	1	2500882	−1	AAACCTAATA**G**GAAAAGGGAC
C7	1	7060076	−1	TGCGGCCTTG**C**GGGAGCAATC
C7	1	23165210	−1	ATCAATGGCT**A**AAAAACCATC
C7	1	30364445	−1	AATGCAAGAG**A**AAGCATTTCA
C7	2	3736597	−1	GTGTATGACC**T**TTATATTTTT
C7	2	16776720	−1	ATCCATAACA**T**TTTTTTTTTG
C7	3	12880551	−1	AGAATGCTCA**T**TTATCTCATT
C7	3	15247466	−1	AAATCATACG**A**CGAACACTAC
C7	4	13256395	−1	TGCTCTTGCC**A**AGGTTAGTTC
C7	5	10287067	−1	CCAAGATCCG**A**ACCTAGAAAT
C7	5	15120725	−1	AATAGATTTC**T**TTTATCGAAA
C7	5	15424664	−1	TGTTTTTTT**G**TGTGTTTTCTT
C116	1	6787418	−1	CAAGAGCGT**C**CACGACGAGTT
C116	1	899668	−1	TGGAGCTGC**T**TCATAAGTTC
C116	2	14954774	−1	AAGCGAAAC**T**TTTATTGCTA
C116	3	3201387	−1	AGCGGTTAT**A**TACATCATAA
C116	5	14227661	−1	TTTTTTTTTC**T**TTTTTTTTAA
C116	5	11032451	−1	CAATTACA**GG**AATGTCGATTT
C197	2	9064276	−1	GAGAACCAAT**C**AAGCCCTAAG
C197	2	17412340	−1	GGAAAGTAAT**A**AGAGCGTTTT
C197	3	15116009	−1	TAGATAAGTA**G**GTTTTTGCGC
C197	4	11372899	−1	AAGAAAATTG**A**ATAGAAAAAA
C352	2	13175805	−1	GTTGCTGCAA**C**CAATAGAGCA
C352	3	20903968	−1	GCGCTTGGAA**T**TTTTTTAATT
C352	3	22734373	−1	AGTGGTTAAA**G**GGTTCCAGCC
C352	4	5256307	−1	CTAAATTCAG**A**AAAAAAAACA
C357	1	24487663	−1	ATGTTACAGT**A**AAAAAAAAAA
C357	2	6789742	−1	ACTAAAGTTG**T**TGCTGCTGAT
C357	3	8430932	−1	ATTCTACTTG**T**CCTCTGAAAT
C357	5	3418139	−1	CACCTCTAAC**A**AAAGTCTTAG
C357	5	7522732	−1	TGAGAATCTC**T**TTTTCTCATT
C357	5	14755177	−1	AAACAATTTC**A**AAGTCCAACC
C541	1	2611420	−1	CCACGTATAT**G**GTAATCACAA
C541	3	5203766	−1	GAAAACTGGT**G**GGAGGTGATC
C541	5	17816447	−1	GACTTGAGAG**T**TTTAACAGAA
C7	3	4723240	−2	GTAGTCATTT**TA**TATATATAGA
C7	4	1435510	−2	AAAACCCCAA**AT**ATAATACTAC
C116	3	4315154	−2	TTTATAACTC**TG**TGCAGTGCTA
C7	2	3072553	−4	AAACGCTCGG**CGAT**GGTGATGATT
C7	3	5701019	−5	TATTAAAAAG**CCAA****C**TTGGTAAAAA
C7	2	8793345	−5	AGCCCATGGA**ATGCT**AATGATTTTG
C116	3	16824813	−5	GGGTGTGAAAA**CTGGT**CAGTTAATAG
C541	1	18202184	−5	TCCTCTCAGT**TCTC****A**TCTGTGGCAT
C7	4	10587368	−6	AGAATTAATC**CACTC****T**TTTTCTTTTT
C7	1	22655118	−6	ACTCCGAAGC**TGTAGA**TGTCTGATTT
C116	1	27885672	−6	CATTTTTATA**TCG****TTT**TGATGGTGAT
C197	1	10625807	−6	TCGGTTGATG**AAGACA**ATGGTAACAT
C197	4	15198376	−7	TTTATCTCCA**AG****TTTTC**CATCTTTCTC
C357	3	442084	−7	CTGAACCTGTG**AACCA**GGAACCCAGTC
C357	5	20249679	−8	ACTTTAAGTT**ACTT****TCTC**ACCAAAAAAA
C197	2	11198490	−9	TTTACTTATG**AATCCTGCT**AATTGAATGAT
C357	1	10405785	−9	TACAGTACAT**AATAA****GTAG**ATAAGTGTAA
C116	3	11700393	−15	CTGCCTTCCCC**C****ATTTGCAGGAC****TTT**CATTGTTATA
C116	1	29852923	−18	GGCCTAGGTA**GATTAAGAGGCTTAA****GCT**GCTGTTGAAT

*Homopolymeric and polynucleotide stretches are underlined; the inserted or deleted base is in bold*.

## Discussion

In this study, we re-sequenced 11 mutagenesis progeny lines (M3) of *A. thaliana* lines derived from CIB irradiation. Based on the obtained data, we could reveal the mutation effects of CIB irradiation on *A. thaliana* at whole genome level, as well as its related molecular mutation spectrum and mutation rates. In contrast to previous studies, we preferred to filter the background mutations shared by multiple re-sequenced lines, rather than rely on the published reference genome. This step was an essential premise for detecting the mutations caused by CIB irradiation. To reduce false positives, we verified the detected mutations by using the IGV, a high-performance visualization tool for the interactive exploration of large, integrated genomic datasets.

Heavy-ion beam irradiation is thought to generate mutations in the form of substitutions, small INDELs and structure variants (i.e., large fragment deletion, inversions, intra-chromosomal translocations, and inter-chromosomal translocations; Tanaka et al., 2010). In this study, we found that the CIB irradiation-induced substitutions prevailed over the INDELs, with the ratio of substitutions to INDELs of 2.58:1 (Figure 5A). *Brachypodium distachyon* mutant line which was induced by chronic gamma radiation, its ratio was 11.90:1 (Lee et al., 2017). As for the fast neutron irradiation, this ratio was 1.45:1 in *A. thaliana*, and 1.26:1 in rice mutant line (Belfield et al., 2012; Li et al., 2016). Among the 124 small INDELs detected here, the number of deletions was more than those of insertions, and the single base INDELs were more prevalent than those in size equal to or greater than 2 bp (Figures 8B,C). Although our current result showed a similar tendency to gamma and fast neutron irradiation (induced more substitution mutations), the proportion of INDELs varied with the quality of radiations. The high LET particles irradiation (i.e., CIB and fast neutron) induced more INDELs than the low LET irradiation. (Belfield et al., 2012; Li et al., 2016; Lee et al., 2017). In fact, we have tried to detect the large INDELs, by simultaneously using both the Pindel (https://trac.nbic.nl/pindel/; Ye et al., 2009) and Break Dancer Max (http://breakdancer.sourceforge.net/breakdancermax.html; Chen et al., 2009). Although hundreds of large deletions were eventually detected (Table S5), most of them were turned out to be false positives after validating them by IGV. This outcome may due to that most commonly used re-sequencing strategy in current is based on the paired-end libraries with an insertion size of 350 bp, for which the read length is 2 × 125 bp rather than the mate pair sequencing libraries whose inserts size can reach up to 2–5 kb. Actually, the long-insert paired-end libraries may be much more suitable for structural variant detection. Secondly, the LET value of the CIB irradiation used in this study was only 50 keV/μm. Previous studies showed that the deletion size and complexity of DNA damage is closely related to the LET value of irradiation (Hada and Georgakilas, 2008; Sage and Harrison, 2011; Hirano et al., 2015). For example, by using a high resolution melting (HRM) technique, CIB irradiation around 30 keV/μm mainly induced substitutions or deletions/insertions, ranging in size from 1 to 53 bp, which was on par with the mutagenesis efficiency of classical chemical mutagen EMS (Kazama et al., 2011). Hirano et al. have reported that heavy-ion beam of 290 keV/μm led to an increased proportion of large deletions (>1 kb) and chromosomal rearrangements (Hirano et al., 2012). Thirdly, the majority of severe DNA damage, including large deletions (from kb to Mb) induced by radiation, cannot be inherited to the offspring, as these non-heritable mutations may be involved in genes that are vital for gamete development or viability. In general, progeny could inherit mutations of 1- or 4-bp deletions (Naito et al., 2005). Considering all these reasons above, to ensure the accuracy and reliability of results in the present study, it was reasonable to focus on substitutions and small INDELs. On the other hand, the mutation rate of the 200-Gy CIB (50 keV/μm) irradiation might be underestimated in this study, because it omitted the detection for the non-transmissible mutations. Our sequencing analyses were based on nine mutagenesis progeny lines (M3) with visible and heritable mutation phenotypes (C7, C116, C197, C352, C357, C541, C600, C828, and C941), as well as two M3 lines with inconspicuous mutation phenotypes (C1001, C1322). In fact, there were still several mutations been detected in C1001, C1322 genomes. This suggests that it is too simple and imprudent to correlate the visible phenotypes to the molecular mutations. Besides, it will underestimate or neglect the potential mutants without eyeable mutation traits. Yan et al. also reported that the effects of the CIB irradiation were underestimated by counting the plants that only displayed abnormal and visible phenotypes (Yan et al., 2014). Otherwise, we previously thought that there might be some mutation hotspot in genome, for example, whether CIB irradiation was prone to induce mutations that were located on certain specific chromosomes? However, based on the variant rate statistics of mutations across each chromosome in the 11 re-sequenced lines, our answer is that so-called hotpots were not observed.

Among the detected substitutions, the ratio of transitions to transversions induced by CIB irradiation was 0.99 in this study. This value differs greatly from the 2.73 of spontaneous substitutions reported in the mutation accumulation line (Ossowski et al., 2010), but closes to the 0.86 reported in a fast neutron-induced mutation line (Belfield et al., 2012). It suggests that artificial mutagenesis can balance the ratio of transitions and transversions. Although an obvious bias of G:C > A:T transitions was observed in this study, compared to mutations induced by EMS which mostly were G:C > A:T substitutions (about 88%), the proportion of this kind of mutations is much lower when treated by CIB irradiation (Henry et al., 2014). For the single base INDELs, however, we found a different bias of A and T bases from the substitutions, and the majority of small INDELs happened at, or adjacent to, homopolymeric or polynucleotide repeats (especially those of A or T bases). Actually, such biases were also prominent in other radiation, such as UV (Daya-Grosjean and Sarasin, 2005) and fast neutron (Belfield et al., 2012). Radiation exposure could induce C > T transitions by causing the formation of covalent linkages between neighboring pyrimidine residues (for instance, CC, CT, TC, and TT) in the DNA sequence, thus resulting in a predominance of UV-induced C > T mutations at the dipyrimidine sequences (Daya-Grosjean and Sarasin, 2005). We had speculated that CIB irradiation-induced C > T mutations likewise followed the mechanisms of preferentially anchoring to pyrimidine dinucleotide sites. By investigating the flanking sequencing of all the 55 C > T mutation sites, 67.27% did occur at the pyrimidine dinucleotide. Given the associations among the UV irradiation, fast neutron, and CIB analyses to date, we infer that radiation of different qualities share a common characteristic of pyrimidine dinucleotide-related C > T transitions. It has been reported that the single base INDELs may be caused by replication slippage at homopolymer or polynucleotide repeat regions (Viguera et al., 2001). To verify this view, the flanking sequence of small INDELs was investigated in the present study. It was found that most of the single base insertions and deletions and the 19 INDELs having sizes ≥2 bp occurred at or close to the homopolymer or polynucleotide repeats. This indicates that CIB irradiation might induce the occurrence of DNA replication slippage.

According to genetic variant annotation and the effect prediction analysis of the 444 detected mutations in the 11 re-sequenced lines, a total of 97 genes—that is to say less than nine genes on average in each genome—incurred relatively high-impact mutations (such as, missense, nonsense, frame shift, or 3′/5′-UTR), which were capable of disrupting the corresponding gene functions. Hence, only a minor proportion of genes would be affected by CIB. Similar results were also observed for Ar-ion (290 keV/μm) and Fe-ion (640 keV/μm) induced mutations (Hirano et al., 2015). This feature possessing by heavy-ions might be a promising tool for plant breeding, because the heavy-ion beam irradiation could alter some characteristics of interest without interfering with other key traits.

Although the main objective of present study is to investigate the mutation spectrum and rate of single base substitutions and small INDELs induced by CIB, it is an essential issue to excavate the mutation resource genes that are associated with plant traits at post-genomics era. Among the 11 re-sequenced lines, C7, C116, C197, C352, C357, C541, C600, C828, and C941 were mutants with stable phenotypes, therefore according to the VarScan 2, homozygous variants were sorted out and performed Gene Ontology (GO) annotation. The detail information of gene ID, family, and biological process involved in were listed in Table S6, the pseudogenes and hypothetic protein genes were filtered out. The rough map based cloning of C197, C352, and C357 were completed in our previous study (Yan et al., 2014). C197 displayed frostbite-like, and uneven leaves. Its mutation sites was located on chromosome 1 (26305380) and chromosome 4 (14740942) respectively, based on the re-sequencing results. AT4G31320, the only gene encoding an SAUR-like auxin-responsive protein family known to be involved in auxin response, was associated with the rough mapping region. C352 displayed an analogous phenotype to the *var2* mutant characterized by a variegated stem, leaves, sepals, and siliques (Takechi et al., 2000). Targeting this trait, we previously performed rough map-based cloning, and the mutated genes were closely linked to the marker T20P8 (Chr 2, 11595846). Fortunately, among the four candidate mutation sites (Figure 4) of C352, one site (chromosome 2, 13175805) with a deletion of a single cytosine which led to the frame shift variant of *var2* gene, was identified. The mutation site of C357 displaying short petiole and compact growth pattern was positioned on the chromosome 3 (989952). Six candidate genes (AT3G02260, AT3G03780, AT3G11540, AT3G12830, AT3G13235, and AT3G44910) were provided by re-sequencing. To ensure the accuracy of results, strict data filtering criterion can minimize the false positive variants. However, it may lead to miss detection. But to a certain extent, the association analysis of rough mapping and whole-genome re-sequencing can provide crucial clues for identifying the corresponding mutations. In fact, to identify gene or locus which are responsible for the interested trait, whole genome re-sequencing analysis, bulked-segregant analysis (BSA), as well as MutMap sequencing strategies have been successfully used for rapidly mapping mutant genes in plant species (Song et al., 2017; Win et al., 2017).

## Conclusion

This study revealed molecular profile and rate of substitutions and small INDELs mutations induced by CIB irradiation in *A. thaliana* at the whole genome level. As heavy-ion beam mutagenesis is an effective and unique mutagen, we will continue our efforts to develop genome sequencing and mutation detection strategies that are more suitable for and more targeted at mutation breeding induced by heavy-ion beam irradiation in the near future. We hope our data could provide valuable clues for explaining the potential mechanism of plant mutation breeding by CIB irradiation.

## Author contributions

LZ coordinated and supervised the project. YD designed the experiments and wrote the manuscript. YD and SL analyzed the data. YD, XL, JY, TC, LY, HF, YC, JM, and XC performed experiments. WJL, QS, TG, and WLL corrected the manuscript. All authors read and approved the final manuscript.

### Conflict of interest statement

The authors declare that the research was conducted in the absence of any commercial or financial relationships that could be construed as a potential conflict of interest.
