# Supplementary material for: Identification of Substitutions and Small Insertion-Deletions Induced by Carbon-Ion Beam Irradiation in Arabidopsis thaliana
Source: Front Plant Sci. 2017 Oct 27;8:1851. doi: 10.3389/fpls.2017.01851 (PMC5665000; doi:10.3389/fpls.2017.01851)
Supplement: Supplementary file 1 [file Table1.DOCX]

**TABLE S1 | Summary of the re-sequencing and mapping results.**

| Line | Total reads | Properly mapped(%)^a^ | Average depth | Cov_ratio_1X(%)^b^ | Cov_ratio_5X(%)^c^ | Cov_ratio_10X(%)^d^ |
| --- | --- | --- | --- | --- | --- | --- |
| Lab-WT | 32996058 | 93.82 | 23 | 99.95 | 99.69 | 97.57 |
| C7 | 39974240 | 92.36 | 29 | 99.95 | 99.8 | 99.08 |
| C116 | 43463880 | 96.97 | 32 | 99.96 | 99.88 | 99.54 |
| C197 | 36306550 | 95.32 | 25 | 99.95 | 99.82 | 99.09 |
| C352 | 35706844 | 95.28 | 23 | 99.95 | 99.77 | 98.57 |
| C357 | 40652752 | 94.36 | 30 | 99.74 | 98.67 | 96.76 |
| C541 | 44200658 | 95.81 | 33 | 99.96 | 99.9 | 99.66 |
| C600 | 40234148 | 95.78 | 31 | 99.96 | 99.87 | 99.54 |
| C828 | 37454892 | 95.92 | 27 | 99.96 | 99.83 | 99.23 |
| C941 | 36791756 | 97.02 | 26 | 99.96 | 99.84 | 99.11 |
| C1001 | 36670782 | 96.2 | 27 | 99.71 | 99.58 | 98.96 |
| C1322 | 39599302 | 94.87 | 29 | 99.96 | 99.86 | 99.38 |
| Average | 38670989 | 95.31 | 27.92 | 99.92 | 99.71 | 98.87 |

*^a^ The properly mapped ratios represents the percentage of reads with both paired-ends mapped onto the reference genome, with a distance that accords with the length distribution of the sequencing fragments.*

*^b, c, d^ The bases with mapping depth greater than 1-, 5-, and 10-fold of the total reference genome on average, respectively.*
