# Supplementary material for: Identification of Substitutions and Small Insertion-Deletions Induced by Carbon-Ion Beam Irradiation in Arabidopsis thaliana
Source: Front Plant Sci. 2017 Oct 27;8:1851. doi: 10.3389/fpls.2017.01851 (PMC5665000; doi:10.3389/fpls.2017.01851)
Supplement: Supplementary file 2 [file Table2.DOCX]

**TABLE S2 | List of mutations predicted to affect gene function in the 11 M3 re-sequenced lines.**

| Line | Mutation type | Chromosome | Loci | Reference base | Variant base | Mutation effect | Gene ID |
| --- | --- | --- | --- | --- | --- | --- | --- |
| C7 | Homozygous | 2 | 8793345 | A | -ATGCT | frameshift_variant | AT2G20370.1 |
|  | Homozygous | 3 | 5701019 | G | -CCAAC | 3_prime_UTR_variant | AT3G16750.1 |
|  | Homozygous | 4 | 12293936 | A | C | missense_variant | AT4G23560.1 |
|  | Homozygous | 4 | 13475278 | T | A | initiator_codon_variant | AT4G26740.1 |
|  | Homozygous | 5 | 16650817 | C | G | missense_variant | AT5G41630.1 |
|  | Homozygous | 5 | 16650819 | C | T | missense_variant | AT5G41630.1 |
|  | Homozygous | 5 | 22684748 | G | T | missense_variant | AT5G56020.1 |
|  | Heterozygous | 1 | 17733894 | A | T | missense_variant | AT1G48090.1 |
|  | Heterozygous | 1 | 17871566 | C | +T | frameshift_variant | AT1G48360.1 |
|  | Heterozygous | 1 | 22655118 | C | -TGTAGA | inframe_deletion | AT1G61400.1 |
|  | Heterozygous | 1 | 27116288 | T | C | missense_variant | AT1G72050.2 |
|  | Heterozygous | 2 | 8793345 | A | -ATGCT | frameshift_variant | AT2G20370.1 |
|  | Heterozygous | 3 | 5701019 | G | -CCAAC | 3_prime_UTR_variant | AT3G16750.1 |
|  | Heterozygous | 4 | 10587368 | A | -CACTCT | 3_prime_UTR_variant | AT4G19420.1 |
|  | Heterozygous | 4 | 13256395 | C | -A | frameshift_variant | AT4G26160.1 |
|  | Heterozygous | 5 | 3829172 | C | T | missense_variant | AT5G11880.1 |
| C116 | Homozygous | 1 | 23632627 | G | C | missense_variant | AT1G63710.1 |
|  | Homozygous | 1 | 29852923 | A | -GATTAAGAGGCTTAAGCT | disruptive_inframe_deletion | AT1G79350.1 |
|  | Homozygous | 1 | 6787418 | T | -C | frameshift_variant | AT1G19630.1 |
|  | Homozygous | 2 | 12030214 | G | A | missense_variant | AT2G28210.1 |
|  | Homozygous | 3 | 21948981 | C | T | missense_variant | AT3G59400.1 |
|  | Homozygous | 3 | 3201387 | T | -A | frameshift_variant | AT3G10330.1 |
|  | Homozygous | 4 | 3922658 | C | A | missense_variant | AT4G06688.1 |
|  | Homozygous | 5 | 21435392 | A | T | missense_variant | AT5G52882.1 |
|  | Heterozygous | 1 | 2394826 | T | G | 3_prime_UTR_variant | AT1G07725.1 |
| C197 | Heterozygous | 5 | 9485606 | T | G | missense_variant | AT5G26960.1 |
|  | Heterozygous | 5 | 16686604 | C | G | missense_variant | AT5G41730.1 |
|  | Heterozygous | 2 | 16166701 | C | G | missense_variant | AT2G38660.2 |
|  | Heterozygous | 5 | 5133036 | C | T | missense_variant | AT5G15730.1 |
|  | Heterozygous | 1 | 10625807 | G | -AAGACA | inframe_deletion | AT1G30200.2 |
|  | Heterozygous | 3 | 18283089 | T | C | 5_prime_UTR_variant | AT3G49307.1 |
|  | Heterozygous | 2 | 10180017 | G | T | 3_prime_UTR_variant | AT2G23910.1 |
| C352 | Homozygous | 1 | 18711883 | T | A | missense_variant | AT1G50500.1 |
|  | Homozygous | 1 | 2528831 | A | T | missense_variant | AT1G08100.1 |
|  | Homozygous | 2 | 13175805 | A | -C | frameshift_variant | AT2G30950.1 |
|  | Homozygous | 3 | 22734373 | A | -G | frameshift_variant | AT3G61430.1 |
| C357 | Homozygous | 1 | 26842125 | G | A | missense_variant | AT1G71220.1 |
|  | Homozygous | 1 | 26842126 | A | T | missense_variant | AT1G71220.1 |
|  | Homozygous | 2 | 7893188 | T | A | missense_variant | AT2G18150.1 |
|  | Homozygous | 2 | 9045207 | C | T | 3_prime_UTR_variant | AT2G21080.1 |
|  | Homozygous | 2 | 6789742 | G | -T | frameshift_variant | AT2G15560.1 |
|  | Homozygous | 3 | 3637399 | C | A | missense_variant | AT3G11540.1 |
|  | Homozygous | 3 | 4273476 | C | G | missense_variant | AT3G13235.1 |
|  | Homozygous | 3 | 4273475 | G | A | missense_variant | AT3G13235.1 |
|  | Homozygous | 3 | 442084 | T | -GAACCAG | frameshift_variant | AT3G02260.1 |
|  | Homozygous | 4 | 17922146 | G | A | missense_variant | AT4G38200.1 |
|  | Homozygous | 5 | 9138044 | G | A | missense_variant | AT5G26150.1 |
|  | Homozygous | 5 | 3418139 | C | -A | frameshift_variant | AT5G10800.1 |
|  | Heterozygous | 1 | 10405785 | T | -AATAAGTAG | inframe_deletion | AT1G29730.1 |
|  | Heterozygous | 1 | 11553334 | G | C | missense_variant | AT1G32120.1 |
|  | Heterozygous | 1 | 19647027 | A | G | missense_variant | AT1G52750.1 |
| C357 | Heterozygous | 3 | 8430932 | G | -T | frameshift_variant | AT3G23510.1 |
|  | Heterozygous | 3 | 21754406 | C | T | missense_variant | AT3G58820.1 |
| C541 | Homozygous | 1 | 18202184 | T | -TCTCA | frameshift_variant | AT1G49210.1 |
|  | Homozygous | 2 | 16964601 | C | A | missense_variant | AT2G40650.1 |
|  | Heterozygous | 1 | 23253938 | G | C | missense_variant | AT1G62800.2 |
|  | Heterozygous | 1 | 24113895 | A | T | stop_gained | AT1G64900.1 |
|  | Heterozygous | 3 | 5203766 | T | -G | frameshift_variant | AT3G15410.1 |
|  | Heterozygous | 5 | 16565496 | C | T | 5_prime_UTR_variant | AT5G41390.1 |
| C600 | Homozygous | 2 | 8141358 | T | A | stop_gained | AT2G18790.1 |
|  | Homozygous | 5 | 4935418 | A | G | 3_prime_UTR_variant | AT5G15200.2 |
|  | Heterozygous | 1 | 4512246 | G | A | missense_variant | AT1G13210.1 |
|  | Heterozygous | 1 | 7278115 | A | -TCAGCTTTCCGTAACTGTTTG | frameshift_variant | AT1G20910.1 |
|  | Heterozygous | 3 | 4820033 | G | A | missense_variant | AT3G14415.1 |
|  | Heterozygous | 4 | 80840 | T | -G | frameshift_variant | AT4G00190.1 |
|  | Heterozygous | 4 | 2725697 | T | G | missense_variant | AT4G05340.1 |
|  | Heterozygous | 4 | 16013237 | C | +TTTT | frameshift_variant | AT4G33200.1 |
|  | Heterozygous | 4 | 16343069 | T | A | 3_prime_UTR_variant | AT4G34120.1 |
|  | Heterozygous | 4 | 12005191 | C | T | missense_variant | AT4G22880.1 |
|  | Heterozygous | 4 | 15015760 | T | -C | frameshift_variant | AT4G30830.1 |
|  | Heterozygous | 5 | 3858834 | C | A | 3_prime_UTR_variant | AT5G11960.1 |
|  | Heterozygous | 5 | 4648997 | T | -C | frameshift_variant | AT5G14420.1 |
|  | Heterozygous | 5 | 16686475 | A | G | missense_variant | AT5G41730.1 |
|  | Heterozygous | 5 | 19571211 | G | T | stop_gained | AT5G48300.1 |
| C828 | Heterozygous | 3 | 8117736 | A | -CC | frameshift_variant | AT3G22910.1 |
|  | Heterozygous | 1 | 6658436 | T | G | missense_variant | AT1G19260.1 |
|  | Heterozygous | 5 | 26829330 | C | -TCT | 3_prime_UTR_variant | AT5G67245.1 |
| C941 | Homozygous | 1 | 7928990 | A | -G | frameshift_variant | AT1G22460.1 |
|  | Homozygous | 2 | 7460595 | A | G | missense_variant | AT2G17120.1 |
|  | Homozygous | 2 | 1519588 | T | G | 3_prime_UTR_variant | AT2G04360.1 |
|  | Homozygous | 4 | 677826 | T | C | missense_variant | AT4G01560.1 |
|  | Homozygous | 4 | 6924288 | C | T | missense_variant | AT4G11380.1 |
|  | Homozygous | 4 | 10053978 | T | A | 3_prime_UTR_variant | AT4G18150.1 |
|  | Heterozygous | 1 | 1797858 | G | A | stop_gained | AT1G05920.1 |
|  | Heterozygous | 2 | 12645936 | C | T | 5_prime_UTR_variant | AT2G29550.1 |
|  | Heterozygous | 3 | 19849619 | C | G | missense_variant | AT3G53540.1 |
|  | Heterozygous | 3 | 19145845 | T | -G | frameshift_variant | AT3G51620.1 |
|  | Heterozygous | 3 | 21200600 | G | C | missense_variant | AT3G57300.1 |
|  | Heterozygous | 5 | 9320502 | T | -TTCTCGAA | frameshift_variant | AT5G26670.1 |
|  | Heterozygous | 5 | 23231735 | A | G | missense_variant | AT5G57350.1 |
| C1001 | Homozygous | 4 | 8980212 | G | -GT | frameshift_variant | AT4G15780.1 |
|  | Homozygous | 4 | 14628773 | A | C | missense_variant | AT4G29910.1 |
|  | Heterozygous | 1 | 5516500 | T | -C | frameshift_variant | AT1G16090.1 |
|  | Heterozygous | 1 | 18997059 | A | G | stop_lost | AT1G51230.1 |
| C1322 | Heterozygous | 4 | 12231123 | A | -G | frameshift_variant | AT4G23430.1 |
|  | Heterozygous | 2 | 15060094 | G | A | missense_variant | AT2G35860.1 |
|  | Heterozygous | 2 | 15981812 | T | A | missense_variant | AT2G38150.1 |
|  | Heterozygous | 3 | 8085436 | C | A | missense_variant | AT3G22840.1 |
|  | Heterozygous | 4 | 980039 | C | T | missense_variant | AT4G02230.1 |
|  | Heterozygous | 4 | 6630801 | C | G | missense_variant | AT4G10770.1 |
|  | Heterozygous | 5 | 22976726 | T | A | missense_variant | AT5G56820.1 |
|  | Heterozygous | 2 | 11519677 | T | G | 3_prime_UTR_variant | AT2G26990.1 |
|  | Heterozygous | 2 | 8613063 | C | T | 5_prime_UTR_variant | AT2G19940.2 |
