# Supplementary material for: Identification of Substitutions and Small Insertion-Deletions Induced by Carbon-Ion Beam Irradiation in Arabidopsis thaliana
Source: Front Plant Sci. 2017 Oct 27;8:1851. doi: 10.3389/fpls.2017.01851 (PMC5665000; doi:10.3389/fpls.2017.01851)
Supplement: Supplementary file 3 [file Table3.DOCX]

**TABLE S3 | Calculated single nucleotide mutation rates of the CIB irradiation on *Arabidopsis thaliana*.**

|  | C7 | C116 | C197 | C352 | C357 | C541 | C600 | C828 | C941 | C1001 | C1322 | Total |
| --- | --- | --- | --- | --- | --- | --- | --- | --- | --- | --- | --- | --- |
| M3 hom SNPs | 12 | 18 | 6 | 14 | 27 | 9 | 14 | 5 | 22 | 3 | 6 | 136 |
| M1 het SNPs | 32.00 | 48.00 | 16.00 | 37.33 | 72.00 | 24.00 | 37.33 | 13.33 | 58.67 | 8.00 | 16.00 | 362.67 |
| M1 carbon ions mutation rate (10×^-7^) | 2.62 | 3.96 | 1.28 | 3.07 | 5.98 | 1.95 | 3.07 | 1.05 | 4.86 | 0.61 | 1.28 | 2.7 |
| M3 hom single base insertions | 2 | 0 | 0 | 0 | 1 | 0 | 1 | 0 | 3 | 0 | 0 | 7 |
| M1 het single base insertions | 5.33 | 0.00 | 0.00 | 0.00 | 2.67 | 0.00 | 2.67 | 0.00 | 8.00 | 0.00 | 0.00 | 18.67 |
| M1 carbon ions mutation rate (10×^-7^) | 0.44 | 0 | 0 | 0 | 0.22 | 0 | 0.22 | 0 | 0.67 | 0 | 0 | 0.14 |
| M3 hom single base deletions | 4 | 3 | 1 | 4 | 3 | 1 | 0 | 0 | 7 | 1 | 2 | 26 |
| M1 het single base deletions | 10.67 | 8.00 | 2.67 | 10.67 | 8.00 | 2.67 | 0.00 | 0.00 | 18.67 | 2.67 | 5.33 | 69.33 |
| M1 carbon ions mutation rate(10×^-7^) | 0.89 | 0.67 | 0.22 | 0.89 | 0.67 | 0.22 | 0 | 0 | 1.56 | 0.22 | 0.45 | 0.53 |

*The M1 heterozygous (het) mutations based on the M3 numbers of homozygous (hom), according to the Mendelian principles (Belfield et al. 2012) and the spontaneous mutations, were corrected by the Col-0 MA line mutation rates (Ossowski et al. 2010).*
