# Supplementary material for: Identification of Substitutions and Small Insertion-Deletions Induced by Carbon-Ion Beam Irradiation in Arabidopsis thaliana
Source: Front Plant Sci. 2017 Oct 27;8:1851. doi: 10.3389/fpls.2017.01851 (PMC5665000; doi:10.3389/fpls.2017.01851)
Supplement: Supplementary file 4 [file Table4.DOCX]

**TABLE S4 | Substitutions identified in 11 M3 lines.**

| Mutation | C7 | C116 | C197 | C352 | C357 | C541 | C600 | C828 | C941 | C1001 | C1322 | Total |
| --- | --- | --- | --- | --- | --- | --- | --- | --- | --- | --- | --- | --- |
| A>C | 4 | 2 | 0 | 0 | 0 | 1 | 1 | 0 | 1 | 1 | 3 | 13 |
| A>G | 1 | 2 | 1 | 1 | 2 | 4 | 5 | 0 | 5 | 1 | 4 | 26 |
| A>T | 8 | 2 | 3 | 3 | 4 | 4 | 1 | 1 | 3 | 2 | 4 | 35 |
| C>A | 1 | 2 | 2 | 0 | 2 | 1 | 2 | 0 | 0 | 3 | 5 | 18 |
| C>G | 2 | 2 | 3 | 0 | 1 | 1 | 0 | 2 | 1 | 0 | 2 | 14 |
| C>T | 4 | 6 | 8 | 2 | 8 | 3 | 5 | 1 | 10 | 3 | 5 | 55 |
| G>A | 2 | 8 | 3 | 3 | 5 | 4 | 6 | 1 | 6 | 3 | 10 | 51 |
| G>C | 0 | 2 | 1 | 0 | 2 | 1 | 0 | 0 | 1 | 0 | 1 | 8 |
| G>T | 3 | 2 | 1 | 0 | 1 | 1 | 4 | 1 | 3 | 2 | 2 | 20 |
| T>A | 3 | 2 | 0 | 2 | 6 | 0 | 6 | 0 | 5 | 2 | 8 | 34 |
| T>C | 1 | 2 | 5 | 2 | 2 | 1 | 7 | 0 | 2 | 1 | 4 | 27 |
| T>G | 2 | 2 | 1 | 2 | 0 | 1 | 3 | 2 | 2 | 0 | 4 | 19 |
