# Supplementary material for: Identification of Substitutions and Small Insertion-Deletions Induced by Carbon-Ion Beam Irradiation in Arabidopsis thaliana
Source: Front Plant Sci. 2017 Oct 27;8:1851. doi: 10.3389/fpls.2017.01851 (PMC5665000; doi:10.3389/fpls.2017.01851)
Supplement: Supplementary file 5 [file Table5.DOCX]

**TABLE S5 | Large deletions detected by Pindel and Break Dancer.**

| Line | Position 1 | Orientation 1^a^ | Position 2 | Orientation 2 | Size |
| --- | --- | --- | --- | --- | --- |
| C7 | chr1:13200894 | 47+47- | chr1:13201697 | 47+47- | 104 |
| C7 | chr1:13992762 | 67+65- | chr1:13993511 | 67+65- | 108 |
| C7 | chr1:14509091 | 49+1- | chr1:14510311 | 6+12- | 1061 |
| C7 | chr1:14511748 | 32+0- | chr1:14538717 | 1+34- | 27001 |
| C7 | chr1:14544675 | 12+0- | chr1:14581720 | 0+52- | 37067 |
| C7 | chr1:14545811 | 61+13- | chr1:14581720 | 0+42- | 38817 |
| C7 | chr1:16510017 | 13+17- | chr1:16510271 | 0+13- | 72 |
| C7 | chr1:16517628 | 16+0- | chr1:16526960 | 0+14- | 9119 |
| C7 | chr1:17245899 | 11+12- | chr1:17246506 | 11+12- | 81 |
| C7 | chr1:17639052 | 28+28- | chr1:17639806 | 28+28- | 109 |
| C7 | chr1:20085588 | 11+0- | chr1:20090242 | 0+13- | 4698 |
| C7 | chr1:24885038 | 74+50- | chr1:24885816 | 74+50- | 85 |
| C7 | chr1:28537145 | 46+52- | chr1:28537913 | 46+52- | 104 |
| C7 | chr3:5281492 | 13+0- | chr3:5282076 | 1+49- | 551 |
| C7 | chr3:9171356 | 31+31- | chr3:9172352 | 31+31- | 104 |
| C7 | chr3:11353781 | 23+22- | chr3:11354593 | 23+22- | 93 |
| C7 | chr3:13022150 | 39+50- | chr3:13023055 | 39+50- | 106 |
| C7 | chr3:14218609 | 26+0- | chr3:14224799 | 30+33- | 6569 |
| C7 | chr4:3056512 | 8+8- | chr4:3057209 | 8+8- | 70 |
| C7 | chr4:3099784 | 9+0- | chr4:3100198 | 0+9- | 309 |
| C7 | chr4:9186974 | 39+16- | chr4:9188005 | 16+41- | 1645 |
| C7 | chr5:4002694 | 20+29- | chr5:4002894 | 8+25- | 74 |
| C7 | chr5:4009021 | 24+6- | chr5:4011941 | 0+8- | 2913 |
| C7 | chr5:13446284 | 13+0- | chr5:13449811 | 0+43- | 3542 |
| C7 | chr5:13570181 | 11+0- | chr5:13570460 | 15+15- | 67 |
| C116 | chr1:13200464 | 63+63- | chr1:13201671 | 63+63- | 103 |
| C116 | chr1:13992773 | 61+60- | chr1:13993506 | 61+60- | 109 |
| C116 | chr1:14511743 | 44+1- | chr1:14538721 | 0+50- | 26996 |
| C116 | chr1:14545812 | 73+10- | chr1:14581719 | 0+64- | 36000 |
| C116 | chr1:15437990 | 144+146- | chr1:15438187 | 0+16- | 856 |
| C116 | chr1:16517628 | 30+0- | chr1:16526780 | 1+26- | 9115 |
| C116 | chr1:17245878 | 15+14- | chr1:17246604 | 15+14- | 82 |
| C116 | chr1:17638594 | 33+31- | chr1:17639793 | 33+31- | 109 |
| C116 | chr1:20085593 | 8+21- | chr1:20090474 | 0+8- | 4710 |
| C116 | chr1:24885036 | 48+53- | chr1:24885683 | 48+53- | 86 |
| C116 | chr1:28537127 | 56+63- | chr1:28537939 | 56+63- | 112 |
| C116 | chr3:5281485 | 17+0- | chr3:5282076 | 2+45- | 551 |
| C116 | chr3:9171551 | 38+36- | chr3:9172340 | 38+36- | 115 |
| C116 | chr3:11353823 | 55+56- | chr3:11354589 | 55+56- | 105 |
| C116 | chr3:13022157 | 50+58- | chr3:13023030 | 50+58- | 106 |
| C116 | chr3:14218620 | 35+0- | chr3:14224761 | 41+46- | 6572 |
| C116 | chr4:2979556 | 10+2- | chr4:2980643 | 4+10- | 1087 |
| C116 | chr4:9187037 | 42+22- | chr4:9188004 | 20+44- | 1643 |
| C116 | chr4:11750915 | 26+13- | chr4:11754866 | 58+68- | 184 |
| C116 | chr5:4009239 | 34+6- | chr5:4011940 | 0+10- | 2917 |
| C116 | chr5:11181329 | 18+12- | chr5:11182075 | 18+12- | 79 |
| C116 | chr5:13446288 | 9+1- | chr5:13449807 | 0+36- | 3527 |
| C116 | chr5:13570224 | 15+3- | chr5:13570461 | 3+24- | 72 |
| C197 | chr1:13200876 | 43+46- | chr1:13201675 | 43+46- | 104 |
| C197 | chr1:13992749 | 46+42- | chr1:13993511 | 46+42- | 107 |
| C197 | chr1:14509042 | 35+0- | chr1:14510311 | 11+16- | 1046 |
| C197 | chr1:14511746 | 33+1- | chr1:14538721 | 2+34- | 27002 |
| C197 | chr1:14545808 | 56+23- | chr1:14581720 | 1+40- | 39220 |
| C197 | chr1:16509942 | 16+43- | chr1:16510369 | 16+43- | 76 |
| C197 | chr1:16517629 | 34+0- | chr1:16526964 | 0+28- | 9130 |
| C197 | chr1:17639057 | 31+30- | chr1:17639777 | 31+30- | 111 |
| C197 | chr1:20085582 | 14+1- | chr1:20090229 | 0+14- | 4703 |
| C197 | chr1:24885038 | 41+44- | chr1:24885678 | 41+44- | 95 |
| C197 | chr1:28537170 | 47+46- | chr1:28538316 | 47+46- | 106 |
| C197 | chr2:2629556 | 24+27- | chr2:2652751 | 18+19- | 23509 |
| C197 | chr3:5281501 | 23+0- | chr3:5282078 | 2+62- | 554 |
| C197 | chr3:9171567 | 40+47- | chr3:9172446 | 40+47- | 121 |
| C197 | chr3:11353810 | 37+40- | chr3:11354594 | 37+40- | 104 |
| C197 | chr3:13022309 | 48+46- | chr3:13023068 | 48+46- | 107 |
| C197 | chr3:13589685 | 106+149- | chr3:13589920 | 3719+1398- | 142 |
| C197 | chr3:14194281 | 24+62- | chr3:14195647 | 24+62- | 79 |
| C197 | chr3:14218639 | 28+3- | chr3:14224742 | 40+33- | 6568 |
| C197 | chr3:14475441 | 29+25- | chr3:14476824 | 29+25- | 93 |
| C197 | chr3:20745476 | 34+0- | chr3:20746223 | 9+34- | 778 |
| C197 | chr3:22476351 | 23+1- | chr3:22477333 | 1+24- | 1056 |
| C197 | chr4:3099770 | 13+2- | chr4:3100227 | 0+13- | 316 |
| C197 | chr4:9186982 | 54+12- | chr4:9188005 | 12+58- | 1648 |
| C197 | chr5:4002683 | 14+7- | chr5:4002888 | 0+25- | 77 |
| C352 | chr1:13200922 | 43+49- | chr1:13201670 | 43+49- | 104 |
| C352 | chr1:13992784 | 50+45- | chr1:13993515 | 50+45- | 107 |
| C352 | chr1:14511741 | 26+0- | chr1:14538721 | 0+37- | 27006 |
| C352 | chr1:14545811 | 56+13- | chr1:14581720 | 0+47- | 38947 |
| C352 | chr1:15437988 | 140+107- | chr1:15438300 | 0+11- | 858 |
| C352 | chr1:16509933 | 21+44- | chr1:16510468 | 21+44- | 68 |
| C352 | chr1:16517626 | 14+0- | chr1:16526961 | 0+11- | 9121 |
| C352 | chr1:17639049 | 31+29- | chr1:17639789 | 31+29- | 110 |
| C352 | chr1:24884895 | 60+55- | chr1:24885684 | 60+55- | 86 |
| C352 | chr1:28537160 | 50+56- | chr1:28537924 | 50+56- | 107 |
| C352 | chr2:2629574 | 31+27- | chr2:2652739 | 20+22- | 23511 |
| C352 | chr3:5281495 | 14+1- | chr3:5282076 | 1+31- | 557 |
| C352 | chr3:9171509 | 38+38- | chr3:9172313 | 38+38- | 115 |
| C352 | chr3:11353810 | 40+39- | chr3:11354579 | 40+39- | 104 |
| C352 | chr3:13022311 | 39+36- | chr3:13023075 | 39+36- | 102 |
| C352 | chr3:14158797 | 2+4- | chr3:14474640 | 59+56- | 87 |
| C352 | chr3:14218618 | 26+0- | chr3:14224528 | 23+31- | 6570 |
| C352 | chr4:3099779 | 10+0- | chr4:3100206 | 0+9- | 322 |
| C352 | chr4:6665866 | 11+9- | chr4:6666581 | 11+9- | 69 |
| C352 | chr4:9186607 | 30+0- | chr4:9188005 | 13+31- | 1644 |
| C352 | chr5:4002686 | 11+7- | chr5:4002894 | 5+32- | 73 |
| C352 | chr5:13446255 | 13+0- | chr5:13449837 | 1+24- | 3542 |
| C357 | chr1:13841221 | 17+15- | chr4:3506215 | 60+59- | 73 |
| C357 | chr1:13200719 | 47+50- | chr1:13201688 | 47+50- | 101 |
| C357 | chr1:13992765 | 67+66- | chr1:13993624 | 67+66- | 108 |
| C357 | chr1:14509042 | 32+1- | chr1:14509412 | 11+47- | 1065 |
| C357 | chr1:14511753 | 45+0- | chr1:14538717 | 1+43- | 27000 |
| C357 | chr1:14545807 | 53+19- | chr1:14581720 | 0+43- | 40077 |
| C357 | chr1:15082323 | 26+52- | chr1:15082650 | 87+189- | 186 |
| C357 | chr1:16509954 | 13+31- | chr1:16510370 | 13+31- | 68 |
| C357 | chr1:16517628 | 21+0- | chr1:16526963 | 0+17- | 9121 |
| C357 | chr1:17639022 | 33+40- | chr1:17639652 | 33+40- | 106 |
| C357 | chr1:20085728 | 8+4- | chr1:20089734 | 6+11- | 4698 |
| C357 | chr1:24885037 | 64+69- | chr1:24886002 | 64+69- | 88 |
| C357 | chr1:28537164 | 48+57- | chr1:28538236 | 48+57- | 106 |
| C357 | chr3:5281501 | 23+1- | chr3:5281940 | 3+59- | 557 |
| C357 | chr3:9171534 | 51+46- | chr3:9172292 | 51+46- | 111 |
| C357 | chr3:13022315 | 57+52- | chr3:13023229 | 57+52- | 106 |
| C357 | chr3:14218608 | 44+1- | chr3:14224808 | 28+55- | 6566 |
| C357 | chr3:14476238 | 24+9- | chr3:14476394 | 18+27- | 86 |
| C357 | chr3:20745744 | 62+3- | chr3:20746162 | 24+58- | 777 |
| C357 | chr4:3099766 | 11+0- | chr4:3100200 | 0+11- | 310 |
| C357 | chr4:9186613 | 53+5- | chr4:9188005 | 1+47- | 1647 |
| C357 | chr5:13446289 | 16+0- | chr5:13449764 | 4+47- | 3539 |
| C357 | chr5:13570197 | 12+3- | chr5:13570461 | 12+16- | 69 |
| C541 | chr1:13200806 | 55+54- | chr1:13201697 | 55+54- | 105 |
| C541 | chr1:13992761 | 62+71- | chr1:13993539 | 62+71- | 109 |
| C541 | chr1:14511750 | 38+0- | chr1:14538719 | 2+39- | 26997 |
| C541 | chr1:14545808 | 78+29- | chr1:14581720 | 1+62- | 38021 |
| C541 | chr1:16510023 | 11+15- | chr1:16510277 | 0+10- | 74 |
| C541 | chr1:16517629 | 27+0- | chr1:16526970 | 2+24- | 9120 |
| C541 | chr1:17638994 | 39+35- | chr1:17640240 | 39+35- | 112 |
| C541 | chr1:20085681 | 11+1- | chr1:20090485 | 2+11- | 4716 |
| C541 | chr1:24885035 | 63+56- | chr1:24885679 | 63+56- | 88 |
| C541 | chr1:28537021 | 45+52- | chr1:28537945 | 45+52- | 111 |
| C541 | chr2:2629578 | 47+48- | chr2:2652750 | 19+29- | 23509 |
| C541 | chr2:3619300 | 3+8- | chr2:3619463 | 16+22- | 79 |
| C541 | chr3:5281501 | 20+0- | chr3:5282078 | 0+55- | 555 |
| C541 | chr3:9171330 | 36+39- | chr3:9172465 | 36+39- | 113 |
| C541 | chr3:11353795 | 47+43- | chr3:11356014 | 47+43- | 109 |
| C541 | chr3:12101827 | 0+10- | chr3:14474735 | 75+70- | 87 |
| C541 | chr3:13022319 | 40+49- | chr3:13023095 | 40+49- | 104 |
| C541 | chr3:14218638 | 34+3- | chr3:14224521 | 35+48- | 6568 |
| C541 | chr3:20745511 | 61+4- | chr3:20746223 | 19+56- | 779 |
| C541 | chr4:2979692 | 11+2- | chr4:2980630 | 5+11- | 1081 |
| C541 | chr4:3054888 | 10+0- | chr4:3055151 | 0+9- | 82 |
| C541 | chr4:3099790 | 19+0- | chr4:3100200 | 1+19- | 317 |
| C541 | chr4:6665821 | 11+11- | chr4:6666903 | 11+11- | 76 |
| C541 | chr4:9187644 | 57+25- | chr4:9188005 | 23+51- | 1642 |
| C541 | chr4:11818120 | 72+4- | chr4:12660874 | 65+82- | 843299 |
| C541 | chr4:12661979 | 56+11- | chr4:13635986 | 26+84- | 974747 |
| C541 | chr5:13446287 | 19+0- | chr5:13449808 | 4+54- | 3535 |
| C600 | chr1:2673699 | 22+47- | chr1:3369930 | 49+24- | 697152 |
| C600 | chr1:13200910 | 72+73- | chr1:13201705 | 72+73- | 106 |
| C600 | chr1:13992603 | 61+60- | chr1:13993844 | 61+60- | 103 |
| C600 | chr1:14511742 | 39+0- | chr1:14538721 | 0+41- | 27002 |
| C600 | chr1:14544719 | 9+1- | chr1:14581720 | 1+61- | 37063 |
| C600 | chr1:14545746 | 59+11- | chr1:14581720 | 1+53- | 35999 |
| C600 | chr1:15437989 | 129+131- | chr1:15438329 | 0+12- | 861 |
| C600 | chr1:16509954 | 10+20- | chr1:16510372 | 10+20- | 74 |
| C600 | chr1:16517692 | 14+1- | chr1:16526973 | 0+10- | 9120 |
| C600 | chr1:17639031 | 44+43- | chr1:17639782 | 44+43- | 111 |
| C600 | chr1:20085584 | 16+14- | chr1:20089709 | 15+16- | 4699 |
| C600 | chr1:24885038 | 58+65- | chr1:24885837 | 58+65- | 89 |
| C600 | chr1:28537142 | 41+47- | chr1:28537989 | 41+47- | 108 |
| C600 | chr2:2629559 | 78+49- | chr2:2652750 | 28+42- | 23506 |
| C600 | chr3:5281501 | 15+3- | chr3:5282077 | 1+53- | 566 |
| C600 | chr3:9171395 | 53+54- | chr3:9172316 | 53+54- | 115 |
| C600 | chr3:11353749 | 41+45- | chr3:11354607 | 41+45- | 106 |
| C600 | chr3:13022330 | 45+47- | chr3:13023325 | 45+47- | 111 |
| C600 | chr3:14204694 | 10+0- | chr3:14204864 | 0+10- | 87 |
| C600 | chr3:14218599 | 36+3- | chr3:14224808 | 36+41- | 6565 |
| C600 | chr3:20745510 | 51+2- | chr3:20746223 | 17+48- | 778 |
| C600 | chr4:3099786 | 21+0- | chr4:3100212 | 0+22- | 315 |
| C600 | chr4:7548835 | 9+10- | chr4:7549522 | 9+10- | 78 |
| C600 | chr4:9186597 | 48+2- | chr4:9188005 | 1+52- | 1645 |
| C600 | chr5:4009015 | 27+1- | chr5:4011943 | 9+10- | 2919 |
| C600 | chr5:13446289 | 10+0- | chr5:13449806 | 1+36- | 3543 |
| C828 | chr1:13200685 | 55+62- | chr1:13201908 | 55+62- | 107 |
| C828 | chr1:13992771 | 33+38- | chr1:13993478 | 33+38- | 108 |
| C828 | chr1:14511775 | 33+0- | chr1:14538721 | 0+33- | 27001 |
| C828 | chr1:14545807 | 47+13- | chr1:14581720 | 0+40- | 36107 |
| C828 | chr1:16517621 | 16+1- | chr1:16526959 | 0+15- | 9113 |
| C828 | chr1:17639000 | 36+39- | chr1:17639812 | 36+39- | 114 |
| C828 | chr1:20085586 | 11+11- | chr1:20090406 | 0+13- | 4698 |
| C828 | chr1:24884789 | 52+65- | chr1:24885840 | 52+65- | 96 |
| C828 | chr1:28536969 | 46+41- | chr1:28537931 | 46+41- | 110 |
| C828 | chr3:5281496 | 19+2- | chr3:5281661 | 2+54- | 559 |
| C828 | chr3:9171535 | 39+37- | chr3:9172521 | 39+37- | 118 |
| C828 | chr3:11353818 | 45+42- | chr3:11354559 | 45+42- | 104 |
| C828 | chr3:13022129 | 40+42- | chr3:13023050 | 40+42- | 105 |
| C828 | chr3:14194139 | 52+74- | chr3:14196037 | 52+74- | 84 |
| C828 | chr3:14218616 | 26+1- | chr3:14224796 | 26+32- | 6562 |
| C828 | chr4:3099792 | 14+1- | chr4:3100201 | 0+14- | 315 |
| C828 | chr4:9187644 | 44+27- | chr4:9188005 | 24+43- | 1639 |
| C828 | chr5:13446248 | 9+0- | chr5:13449810 | 1+32- | 3548 |
| C941 | chr1:13200939 | 45+51- | chr1:13201703 | 45+51- | 104 |
| C941 | chr1:13992773 | 55+51- | chr1:13993515 | 55+51- | 105 |
| C941 | chr1:14511752 | 38+1- | chr1:14538721 | 3+41- | 27000 |
| C941 | chr1:14545800 | 45+10- | chr1:14581720 | 0+37- | 38371 |
| C941 | chr1:16510027 | 13+11- | chr1:16510282 | 0+13- | 74 |
| C941 | chr1:16517629 | 23+0- | chr1:16526960 | 0+15- | 9127 |
| C941 | chr1:17141645 | 9+5- | chr1:17143540 | 0+33- | 1720 |
| C941 | chr1:17639041 | 40+40- | chr1:17639825 | 40+40- | 109 |
| C941 | chr1:24885038 | 51+57- | chr1:24885868 | 51+57- | 84 |
| C941 | chr1:28537131 | 37+36- | chr1:28537897 | 37+36- | 110 |
| C941 | chr2:15152345 | 63+3- | chr2:16001631 | 57+69- | 849851 |
| C941 | chr3:5281484 | 15+1- | chr3:5282078 | 0+39- | 555 |
| C941 | chr3:9171581 | 39+33- | chr3:9172317 | 39+33- | 120 |
| C941 | chr3:11353813 | 37+39- | chr3:11354702 | 37+39- | 102 |
| C941 | chr3:13022341 | 35+38- | chr3:13023051 | 35+38- | 108 |
| C941 | chr3:14218607 | 30+0- | chr3:14224799 | 19+39- | 6561 |
| C941 | chr4:9186542 | 38+1- | chr4:9188005 | 12+43- | 1642 |
| C941 | chr5:11181333 | 19+10- | chr5:11182081 | 19+10- | 72 |
| C941 | chr5:13446284 | 13+0- | chr5:13449824 | 0+34- | 3546 |
| C941 | chr5:13570223 | 19+0- | chr5:13570388 | 4+17- | 76 |
| C1001 | chr1:13200929 | 48+50- | chr1:13201680 | 48+50- | 102 |
| C1001 | chr1:13992779 | 56+50- | chr1:13993524 | 56+50- | 112 |
| C1001 | chr1:14509090 | 28+1- | chr1:14510311 | 9+10- | 1047 |
| C1001 | chr1:14511728 | 45+0- | chr1:14538721 | 0+46- | 27001 |
| C1001 | chr1:14545804 | 57+14- | chr1:14581720 | 0+50- | 38084 |
| C1001 | chr1:15082323 | 29+49- | chr1:15082650 | 64+163- | 176 |
| C1001 | chr1:16517655 | 15+0- | chr1:16526968 | 0+9- | 9128 |
| C1001 | chr1:17639054 | 37+40- | chr1:17639796 | 37+40- | 107 |
| C1001 | chr1:24885038 | 50+64- | chr1:24885683 | 50+64- | 82 |
| C1001 | chr1:28537172 | 51+62- | chr1:28537917 | 51+62- | 109 |
| C1001 | chr2:2629571 | 36+26- | chr2:2652751 | 22+18- | 23506 |
| C1001 | chr2:9532601 | 47+4- | chr2:9564954 | 3+52- | 32446 |
| C1001 | chr3:5281480 | 11+0- | chr3:5282078 | 0+40- | 548 |
| C1001 | chr3:9171538 | 53+50- | chr3:9172327 | 53+50- | 113 |
| C1001 | chr3:11353826 | 29+36- | chr3:11354593 | 29+36- | 99 |
| C1001 | chr3:13022295 | 55+56- | chr3:13023083 | 55+56- | 106 |
| C1001 | chr3:14158797 | 3+1- | chr3:14475106 | 49+44- | 83 |
| C1001 | chr3:14204496 | 11+10- | chr3:14205104 | 11+10- | 78 |
| C1001 | chr3:14218614 | 32+0- | chr3:14224393 | 29+41- | 6565 |
| C1001 | chr3:14474940 | 17+22- | chr3:14475106 | 28+25- | 70 |
| C1001 | chr4:9186978 | 35+10- | chr4:9188005 | 11+35- | 1648 |
| C1001 | chr4:11750915 | 29+18- | chr4:11754866 | 65+71- | 182 |
| C1001 | chr5:4002685 | 16+23- | chr5:4002894 | 7+34- | 78 |
| C1001 | chr5:4009137 | 36+6- | chr5:4011931 | 2+10- | 2917 |
| C1001 | chr5:11181376 | 14+9- | chr5:11182067 | 14+9- | 73 |
| C1322 | chr1:13200701 | 50+49- | chr1:13201853 | 50+49- | 103 |
| C1322 | chr1:13992394 | 67+63- | chr1:13993549 | 67+63- | 106 |
| C1322 | chr1:14509044 | 37+0- | chr1:14510290 | 7+11- | 1060 |
| C1322 | chr1:14511750 | 37+0- | chr1:14538721 | 1+43- | 27002 |
| C1322 | chr1:14544557 | 10+0- | chr1:14581720 | 1+53- | 37069 |
| C1322 | chr1:14545812 | 49+17- | chr1:14581720 | 1+43- | 39676 |
| C1322 | chr1:16517694 | 26+1- | chr1:16526721 | 1+20- | 9125 |
| C1322 | chr1:17638840 | 41+39- | chr1:17639769 | 41+39- | 115 |
| C1322 | chr1:20085593 | 13+7- | chr1:20090473 | 1+12- | 4705 |
| C1322 | chr1:24885029 | 64+49- | chr1:24885658 | 64+49- | 83 |
| C1322 | chr1:25380904 | 10+11- | chr1:25381621 | 10+11- | 84 |
| C1322 | chr1:28387020 | 17+18- | chr1:28387977 | 17+18- | 84 |
| C1322 | chr1:28536926 | 50+59- | chr1:28538078 | 50+59- | 105 |
| C1322 | chr2:2629519 | 36+32- | chr2:2652750 | 25+24- | 23515 |
| C1322 | chr3:5281493 | 13+0- | chr3:5282078 | 1+52- | 554 |
| C1322 | chr3:9171446 | 44+44- | chr3:9172308 | 44+44- | 120 |
| C1322 | chr3:11353795 | 61+70- | chr3:11354594 | 61+70- | 103 |
| C1322 | chr3:13021904 | 48+51- | chr3:13023274 | 48+51- | 107 |
| C1322 | chr3:13589685 | 85+117- | chr3:13589878 | 2920+893- | 217 |
| C1322 | chr3:14158797 | 8+5- | chr3:14475106 | 55+46- | 83 |
| C1322 | chr3:14218689 | 33+1- | chr3:14224806 | 30+45- | 6572 |
| C1322 | chr4:3099778 | 21+0- | chr4:3100200 | 0+21- | 313 |
| C1322 | chr4:9187062 | 40+22- | chr4:9188005 | 16+37- | 1637 |
| C1322 | chr5:4008990 | 24+1- | chr5:4011943 | 0+9- | 2917 |
| C1322 | chr5:13446281 | 14+0- | chr5:13449802 | 0+42- | 3541 |

*^a^ The number of supporting read pairs that at breakpoint 1; ‘+’, forward direction; ‘-’, negative direction.*
