# Supplementary material for: Identification of Substitutions and Small Insertion-Deletions Induced by Carbon-Ion Beam Irradiation in Arabidopsis thaliana
Source: Front Plant Sci. 2017 Oct 27;8:1851. doi: 10.3389/fpls.2017.01851 (PMC5665000; doi:10.3389/fpls.2017.01851)
Supplement: Supplementary file 6 [file Table6.DOCX]

**TABLE S6 | GO annotation of homozygous variant genes in nine M3 lines that displayed stable mutant phenotypes.**

| Line | Gene ID | Family/Subfamily | Function / involved in |
| --- | --- | --- | --- |
| C7 | AT2G20370 | Xyloglucan galactosyltransferase MUR3 | endomembrane system organization, fucose biosynthetic process, salicylic acid mediated signaling pathway, unidimensional cell growth, xyloglucan biosynthetic process |
|  | AT2G46670 | CCT motif family protein | [function unknown](http://www.arabidopsis.org/servlets/TairObject?type=keyword&id=3226) |
|  | AT3G14205 | Phosphoinositide phosphatase SAC2 | phosphatidylinositol-3-phosphate biosynthetic process, vacuole organization |
|  | AT3G28930 | Protein AIG2 | [response to bacterium](http://www.arabidopsis.org/servlets/TairObject?type=keyword&id=7120) |
|  | AT3G31900 | ATP-dependent helicase family protein | unknown |
|  | AT3G43300 | Immunity associated protein targeted by HopM1 | defense response to bacterium, growth, vesicle-mediated transport |
|  | AT4G00550 | Digalactosyldiacylglycerol synthase 2, chloroplastic | cellular response to phosphate starvation, galactolipid biosynthetic process, glycolipid biosynthetic process |
|  | AT4G03270 | Putative cyclin-D6-1 | cell division, regulation of cell cycle |
|  | AT4G23560 | Glycosyl hydrolase 9B15 | cell wall organization, cellulose catabolic process |
|  | AT4G26740 | Peroxygenase 1 | defense response, embryo development ending in seed dormancy, lipid particle organization, oxidation-reduction process |
|  | AT5G04940 | SU(VAR)3-9 homolog | histone lysine methylation, regulation of gene expression, epigenetic |
|  | AT5G37960 | GroES-like family protein | oxidation reduction, N-terminal protein myristoylation |
|  | AT5G38510 | Rhomboid-related intramembrane serine protease family protein | proteolysis |
|  | AT5G39790 | Chloroplast localized protein PTST | regulation of starch biosynthetic process, starch biosynthetic process |
|  | AT5G41630 | F-box/RNI-like superfamily protein | Unknown |
|  | AT5G56020 | Got1/Sft2-like vescicle transport protein | [protein transport](http://www.arabidopsis.org/servlets/TairObject?type=keyword&id=6910) |
| C116 | AT1G19630 | Cytochrome P450, CYP722A | oxidation reduction |
|  | AT1G63710 | Cytochrome P450,CYP86A7 | fatty acid metabolic process |
|  | AT1G79350 | RING/FYVE/PHD zinc finger domain-containing protein | regulation of transcription, DNA-dependent, embryo development ending in seed dormancy |
|  | AT2G03090 | Expansin-A15 | plant-type cell wall modification, multidimensional cell growth, syncytium formation, unidimensional cell growth |
|  | AT2G04038 | BZIP protein (AtbZIP48) | regulation of transcription |
|  | AT2G28210 | Alpha carbonic anhydrase 2 | response to carbon dioxide, one-carbon metabolic process |
|  | AT3G10330 | Transcription initiation factor IIB-2 | translational initiation, regulation of transcription |
|  | AT3G13320 | Vacuolar cation/proton exchanger 2 | cadmium ion transport |
|  | AT3G27940 | LOB domain-containing protein 26 | unknown |
|  | AT3G45790 | Protein kinase-related protein | protein amino acid phosphorylation |
|  | AT3G59400 | Genomes uncoupled 4, GUN4 | chlorophyll biosynthetic process, chloroplast-nucleus signaling pathway, |
|  | AT3G61300 | C2 calcium/lipid-binding plant phosphoribosyltransferase family protein | tryptophan biosynthetic process |
|  | AT4G02790 | GTP-binding family protein | unknown |
|  | AT4G03210 | Xyloglucan endotransglucosylase/hydrolase protein 9 | carbohydrate metabolic process, cellular glucan metabolic process |
|  | AT4G07390 | Mannose-P-dolichol utilization defect 1 protein homolog 2 | transport |
|  | AT4G08540 | DNA-directed RNA polymerase II protein | positive regulation of autophagy |
|  | AT4G11040 | Protein phosphatase 2C family | protein serine/threonine phosphatase activity |
|  | AT4G34710 | Arginine decarboxylase 2 | stress-inducible (osmotic stress). Double mutant analysis showed that ADC genes are essential for the production of PA, and are required for normal seed development. |
|  | AT5G24900 | Cytochrome P450 714A2 | oxidation reduction |
|  | AT5G36150 | Pentacyclic triterpene synthase 3 | pentacyclic triterpenoid biosynthetic process |
|  | AT5G52882 | P-loop containing nucleoside triphosphate hydrolases superfamily | nucleoside-triphosphatase activity, nucleotide binding, ATP binding |
| C197 | AT2G38580 | Mitochondrial ATP synthase D chain-related protein | unknown |
|  | AT3G58760 | Integrin-linked protein kinase family protein | regulation of signal transduction, protein amino acid phosphorylation |
|  | AT4G31320 | SAUR-like auxin-responsive protein family | auxin response |
| C352 | AT1G08100 | High-affinity nitrate transporter 2.2 | lateral root development, nitrate transport, transmembrane transport |
|  | AT1G50500 | Membrane trafficking VPS53 family | retrograde trafficking of vesicles to the late Golgi |
|  | AT1G68710 | ATPase E1-E2 type family protein | phospholipid transport |
|  | AT1G80740 | DNA (cytosine-5)-methyltransferase | DNA mediated transformation, DNA methylation |
|  | AT2G01900 | DNAse I-like superfamily protein | biological_process unknown |
|  | AT2G30950 | FtsH extracellular protease family | photoinhibition, oxygen and reactive oxygen species metabolic process, thylakoid membrane organization, PSII associated light-harvesting complex II catabolic process, protein catabolic process |
|  | AT3G56380 | Two-component response regulator ARR17 | signal transduction. |
|  | AT3G61430 | Plasma membrane intrinsic protein 1A | response to water deprivation, response to salt stress, transport, water transport |
|  | AT4G08310 | DNA ligase | unknown |
|  | AT4G14790 | ATP-dependent RNA helicase | RNA catabolic process,pollen development,megagametogenesis |
|  | AT4G36220 | Cytochrome P450 84A1 | lignin biosynthesis |
| C357 | AT1G14910 | ENTH/ANTH/VHS superfamily protein | N-terminal protein myristoylation, clathrin coat assembly |
|  | AT1G36730 | Translation initiation factor IF2/IF5 | translational initiation, regulation of translational initiation |
|  | AT1G44920 | Transmembrane protein | unknown |
|  | AT1G58270 | TRAF-like family protein | unknown |
|  | AT1G59720 | Tetratricopeptide repeat (TPR)-like superfamily protein | chloroplast RNA modification and processing,mRNA modification and processing |
|  | AT1G71220 | UDP-glucose:glycoprotein glucosyltransferase | anthocyanin-containing compound metabolic process, carbohydrate biosynthetic process, defense response signaling pathway, resistance gene-independent, plant-type hypersensitive response, response to salicylic acid |
|  | AT2G04034 | Defensin-like (DEFL) family protein | defense response to fungus, killing of cells of other organism |
|  | AT2G15560 | Endonuclease or glycosyl hydrolase | involved in regulation of gene expression, response to oxidative stress |
|  | AT2G18150 | Peroxidase superfamily protein | response to oxidative stress, oxidation reduction, response to nematode |
|  | AT2G21080 | Ras guanine nucleotide exchange factor K | unknown |
|  | AT2G40030 | Nuclear RNA polymerase D1B | defense response to fungus, DNA methylation, posttranscriptional gene silencing by RNA |
|  | AT3G02260 | Auxin transport protein BIG | response to auxin stimulus, auxin polar transport, indeterminate inflorescence morphogenesis, photomorphogenesis, root development |
|  | AT3G03780 | Cytosolic methionine synthase | response to salt stress, methionine biosynthetic process |
|  | AT3G11540 | N-acetyl glucosamine transferase | cell differentiation, flower development, cytokinin-activated and gibberellic acid mediated signaling pathway |
|  | AT3G12830 | Auxin-responsive protein SAUR72 | auxin-activated signaling pathway, multicellular organism development, regulation of growth, response to auxin |
|  | AT3G13235 | Ubiquitin family protein | response to cadmium ion, proteolysis |
|  | AT3G44910 | Cation/H(+) antiporter 12 | cation transport, potassium ion transport, regulation of pH |
|  | AT4G38200 | SEC7-like guanine nucleotide exchange family protein | regulation of ARF protein signal transduction |
|  | AT5G07770 | Formin-like protein 16 | cellular component organization, actin cytoskeleton organization |
|  | AT5G10800 | RNA recognition motif (RRM)-containing protein | RNA processing |
|  | AT5G17930 | MIF4G domain and MA3 domain-containing protein | RNA metabolic process |
|  | AT5G22630 | Arogenate dehydratase 5 | L-phenylalanine biosynthetic process |
|  | AT5G26150 | Protein kinase | protein amino acid phosphorylation, response to stress |
|  | AT5G49820 | root UVB sensitive protein | embryo development ending in seed dormancy |
| C541 | AT1G01180 | S-adenosyl-L-methionine-dependent methyltransferase domain-containing protein | lipid biosynthetic process |
|  | AT1G49210 | RING/U-box superfamily protein | protein ubiquitination |
|  | AT2G20850 | Protein STRUBBELIG-RECEPTOR FAMILY 1 | protein amino acid phosphorylation |
|  | AT2G40650 | PRP38 family protein | RNA processing,RNA splicing |
|  | AT2G43160 | ENTH/VHS family protein | protein transport |
|  | AT5G34581 | Hydroxyproline-rich glycoprotein family protein | unknown |
|  | AT5G65240 | Leucine-rich repeat protein kinase family protein | protein amino acid phosphorylation |
| C600 | AT1G08630 | threonine aldolase 1 | glycine biosynthetic process,threonine catabolic process |
|  | AT1G19100 | Histidine kinase-, DNA gyrase B-, and HSP90-like ATPase family protein | DNA repair, RNA-directed DNA methylation, defense response, regulation of chromatin silencing |
|  | AT2G02410 | yacP-like NYN domain protein | unknown |
|  | AT2G07180 | Protein kinase superfamily protein | protein amino acid phosphorylation,defense response |
|  | AT2G18790 | Phytochrome B | the light-promotion of seed germination and in the shade avoidance response |
|  | AT3G11700 | Fasciclin-like arabinogalactan protein 18 | response to cyclopentenone, cell adhesion |
|  | AT4G08140 | 26S proteasome non-ATPase regulatory subunit-like protein | regulation of protein catabolic process |
|  | AT4G22560 | Sulfated surface-like glycoprotein | unknown |
|  | AT5G15200 | 40S ribosomal protein S9-1 | translation |
| C828 | AT2G29090 | Abscisic acid 8'-hydroxylase 2 | ABA catabolism |
|  | AT2G36300 | Protein YIPF | unknown |
|  | AT2G40050 | Cysteine/histidine-rich C1 domain-containing protein | zinc ion binding |
|  | AT3G14040 | Pectin lyase-like superfamily protein | involved in carbohydrate metabolic process, cell wall organization |
|  | AT4G15780 | Vesicle-associated membrane protein 724 | transport, vesicle-mediated transport |
|  | AT5G01820 | CBL-interacting serine/threonine-protein kinase | signal transduction, protein amino acid phosphorylation |
| C941 | AT1G22460 | O-fucosyltransferase family protein | transferase activity, transferring glycosyl groups |
|  | AT1G31360 | RECQ helicase L2 | mediate branch migration of Holliday junctions |
|  | AT1G50940 | Electron transfer flavoprotein subunit alpha | copper ion binding |
|  | AT1G53990 | GDSL-lipase 3 | glycerol biosynthetic process, lipid metabolic process |
|  | AT2G04360 | Transmembrane protein | unknown |
|  | AT2G13820 | Bifunctional inhibitor/lipid-transfer protein/seed storage 2S albumin superfamily protein | lipid transport |
|  | AT2G17120 | LysM domain-containing GPI-anchored protein 2 | cell wall macromolecule catabolic process |
|  | AT2G18890 | Protein kinase superfamily protein | protein amino acid phosphorylation |
|  | AT2G39810 | E3 ubiquitin-protein ligase HOS1 | response to cold, negative regulation of transcription, protein ubiquitination |
|  | AT3G52470 | Late embryogenesis abundant hydroxyproline-rich glycoprotein | unknown |
|  | AT3G59790 | Mitogen-activated protein kinase 10 | auxin transport, regulation of gene expression, signal transduction |
|  | AT4G01560 | Ribosomal RNA processing Brix domain protein, | involved in embryo development ending in seed dormancy, xylem and phloem pattern formation |
|  | AT4G09150 | T-complex protein 11 | phosphopantetheine binding |
|  | AT4G11160 | Translation initiation factor 2, small GTP-binding protein | translational initiation |
|  | AT4G11380 | Adaptin family protein | intracellular protein transport, vesicle-mediated transport, protein transport |
|  | AT4G13780 | Methionine-tRNA ligase | response to cadmium ion, methionyl-tRNA aminoacylation |
|  | AT4G25560 | R2R3-MYB transcription factor LAF1 | cell differentiation, far-red light signaling pathway, positive regulation of transcription, regulation of transcription from RNA polymerase II promoter |
|  | AT4G36690 | Splicing factor U2af large subunit A | nuclear mRNA splicing, via spliceosome, defense response to bacterium |
|  | AT4G37850 | Basic helix-loop-helix (bHLH) DNA-binding superfamily protein | regulation of transcription |
|  | AT5G02050 | Mitochondrial glycoprotein family protein | unknown |
|  | AT5G18910 | Protein kinase superfamily protein | protein autophosphorylation |
|  | AT5G37130 | Tetratricopeptide repeat domain-containing protein | binding |
|  | AT5G54060 | Anthocyanin 3-O-glucoside: 2"-O-xylosyl-transferase | anthocyanin modification |
